# Genome-wide cross-trait analysis and Mendelian randomization reveal a shared genetic etiology and causality between COVID-19 and venous thromboembolism

**DOI:** 10.1038/s42003-023-04805-2

**Published:** 2023-04-21

**Authors:** Xin Huang, Minhao Yao, Peixin Tian, Jason Y. Y. Wong, Zilin Li, Zhonghua Liu, Jie V. Zhao

**Affiliations:** 1 School of Public Health, Li Ka Shing Faculty of Medicine, The University of Hong Kong, Pokfulam Road, Hong Kong SAR, China; 2 Department of Statistics and Actuarial Science, The University of Hong Kong, Pokfulam Road, Hong Kong SAR, China; 3 Epidemiology and Community Health Branch, National Heart Lung and Blood Institute, Bethesda, MD USA; 4 Department of Biostatistics and Health Data Science, Indiana University School of Medicine, Indianapolis, IN USA; 5 Department of Biostatistics, Columbia University, New York, NY USA

**Keywords:** Cardiology, Genetics

## Abstract

Venous thromboembolism occurs in up to one-third of patients with COVID-19. Venous thromboembolism and COVID-19 may share a common genetic architecture, which has not been clarified. To fill this gap, we leverage summary-level genetic data from the latest COVID‐19 host genetics consortium and UK Biobank and examine the shared genetic etiology and causal relationship between COVID-19 and venous thromboembolism. The cross-trait and co-localization analyses identify 2, 3, and 4 shared loci between venous thromboembolism and severe COVID-19, COVID-19 hospitalization, SARS-CoV-2 infection respectively, which are mapped to *ABO, ADAMTS13*, *FUT2* genes involved in coagulation functions. Enrichment analysis supports shared biological processes between COVID-19 and venous thromboembolism related to coagulation and immunity. Bi-directional Mendelian randomization suggests that venous thromboembolism was associated with higher risk of three COVID-19 traits, and SARS-CoV-2 infection was associated with a higher risk of venous thromboembolism. Our study provides timely evidence for the genetic etiology between COVID-19 and venous thromboembolism (VTE). Our findings contribute to the understanding of COVID-19 and VTE etiology and provide insights into the prevention and comorbidity management of COVID-19.

## Abstract (web summary)

Joint analysis of existing summary statistics data using genome-wide crosstrait analysis and bi-directional Mendelian randomization provides further evidence for a shared genetic etiology between COVID-19 and venous thromboembolism.

## Introduction

Coronavirus disease 2019 (COVID-19), caused by the severe acute respiratory syndrome coronavirus 2 (SARS-CoV-2) infection, has led to a worldwide pandemic since March 2020, and caused repeated waves of outbreaks across the globe. Venous thromboembolism (VTE) is one common and serious comorbidity. Notably, thrombotic events occur in up to one-third of patients with COVID-19^1^. People with more pronounced thrombotic symptoms are more likely to develop severe COVID-19^2^, and thrombotic complications are a well-established predictor of mortality in people with COVID-19^1^. Consistently, several genome-wide association studies (GWASs) of COVID-19-related traits (severe COVID-19, COVID-19 hospitalization, and SARS-CoV-2 infection) have identified genetic loci in *ABO*^3–6^, an established gene related to thrombosis. This evidence suggests that VTE and COVID-19 may share common genetic architecture. Identifying shared genetic factors contributing to both COVID-19 and VTE can provide novel insights into disease pathogenesis and pinpoint targets for therapeutic development or drug repurposing. However, to our knowledge, this problem has not been comprehensively investigated.

Meanwhile, the causal relationship between VTE and COVID-19 has not been clarified. On the one hand, VTE predicts COVID-19 severity and mortality^1,2^. On the other hand, the cytokine storm and excessive inflammation caused by SARS-CoV-2 infection are hypothesized to lead to systemic coagulation dysfunction;^7–10^ therefore, SARS-CoV-2 infection may increase the risk of VTE. Many institutional evidence-based guidelines supported the use of prophylactic treatments such as anticoagulants for thromboprophylaxis in COVID-19 patients^11^. However, the recommendation is mainly based on observational studies, which might be subject to unmeasured confounding bias. To resolve this issue, Mendelian randomization (MR) study uses genetic variants as instruments for causal inference and can provide unbiased causal effect estimates even in the presence of unmeasured confounding^12^. One previous MR study in a relatively smaller GWAS for COVID-19 suggested that genetically predicted VTE is associated with a higher risk of COVID-19 hospitalization and SARS-CoV-2 infection^13^, however, the association with severe COVID-19 is uncertain possibly due to the limited sample size. The reverse association, i.e., the association of COVID-19 with VTE has not been examined. The recently updated GWAS for COVID-19 with doubled sample size provided a well-powered study dataset to investigate the relationship between VTE and COVID-19.

Taken together, to fill this knowledge gap, we performed a genome-wide cross-trait analysis and colocalization analysis based on summary statistics from GWASs of VTE and COVID-19 and identified shared loci between VTE and three COVID-19-related traits. We also conducted an enrichment analysis and found the shared genes were enriched for expression in the lung tissue and involved in coagulation and immunity. We also applied bi-directional Mendelian randomization to study the causal relationship between VTE and three COVID-19-related traits.

## Results

### Genetic correlation of VTE with COVID-19-related traits

**Fig. 1:**
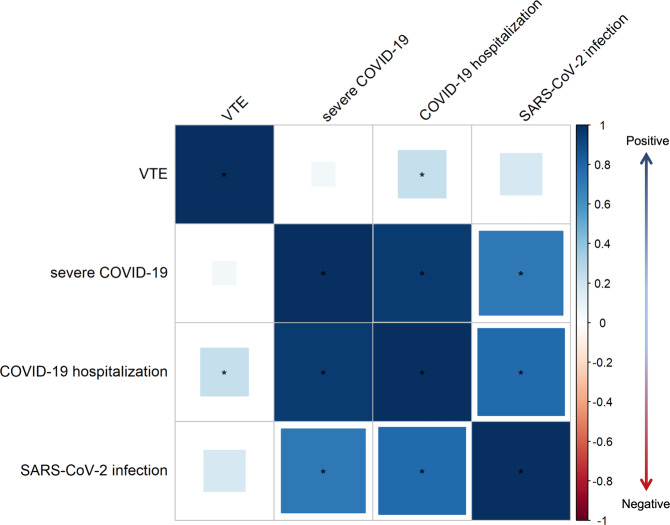
Genetic correlation of VTE with COVID-19-related traits. Results were derived from linkage disequilibrium score regression (LDSC) analysis. Vertical axis represents the genetic correlation estimate Rg, with red indicating a negative correlation and blue indicating a positive correlation. The size of the colored squares is proportional to the *P* value, where larger squares represent a smaller *P* value. Asterisk represents significance (*P* < 0.05). The presented data are available in Supplementary Table 2.

**Fig. 2:**
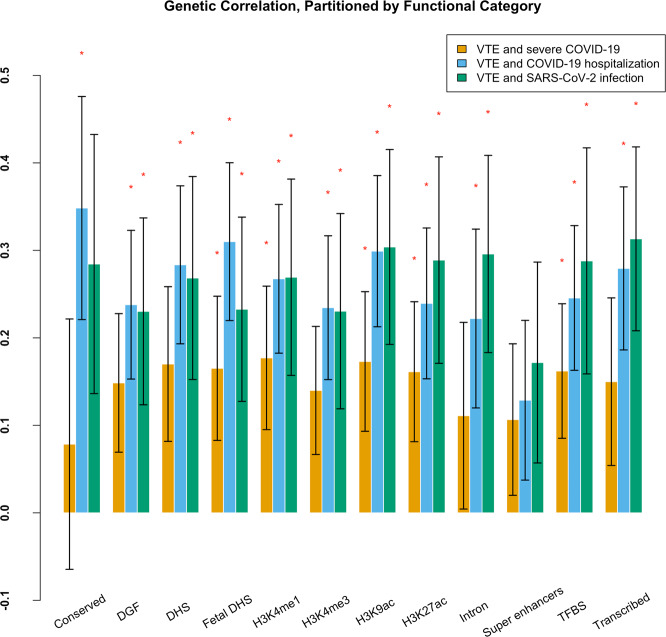
Partitioned genetic correlation between VTE and COVID-19-related traits. Results were derived from partitioned linkage disequilibrium score regression (LDSC) analysis. The *x* axis represents the 12 functional categories, and the *y* axis represents the estimated partitioned genetic correlation. The significant functional categories (*P* < 0.05) are starred. Error bars represent the standard error of genetic correlation estimates. DGF DNaseI digital genomic footprinting, DHS DNase I hypersensitivity site, TFBS transcription factor binding sites. The presented data are available in Supplementary Data 7.

The heritability (*h*^2^) estimated by Linkage disequilibrium score regression (LDSC) analysis suggested severe COVID-19, COVID-19 hospitalization, SARS-CoV-2 infection, and VTE are heritable (*P* < 0.05 shown in Supplementary Table 1). We found positive overall genetic correlation of VTE with COVID-19 hospitalization (*r*_g_ = 0.2320, *P* value = 0.0092) (shown in Fig. 1 and Supplementary Table 2). The genetic correlation between VTE and severe COVID-19 and SARS-CoV-2 was positive but not significant at *p* value of 0.05 level. Further partitioned LDSC analysis found that severe COVID-19, COVID-19 hospitalization, and SARS-CoV-2 infection are genetically correlated with VTE in five (Fetal DNase I hypersensitivity sites (DHS), H3K4me1, H3K9ac H3K27ac and transcription factor-binding site (TFBS)), 11 (except for super-enhancers regions) and 10 (except for conserved and super-enhancers regions) of the 12 functional categories, respectively (Fig. 2 and Supplementary Data 7).

### Multi-trait analysis of GWAS (MTAG)

Based on the COVID-19 host genetics initiative (HGI) updated GWAS meta-analysis (release 7), there were 45 genome-wide significant (*P* < 5 × 10^−8^) and uncorrelated (*r*^2^ < 0.01) loci for severe COVID-19, 46 for COVID-19 hospitalization and 26 for SARS-CoV-2 infection (Supplementary Data 1), and 12 for VTE in the GWAS from UK Biobank (Supplementary Data 2). Compared with these previously identified SNPs, using MTAG incorporating information from GWAS of COVID-19 and VTE we did not identify additional genome-wide significant loci for COVID-19 traits or VTE.

**Fig. 3:**
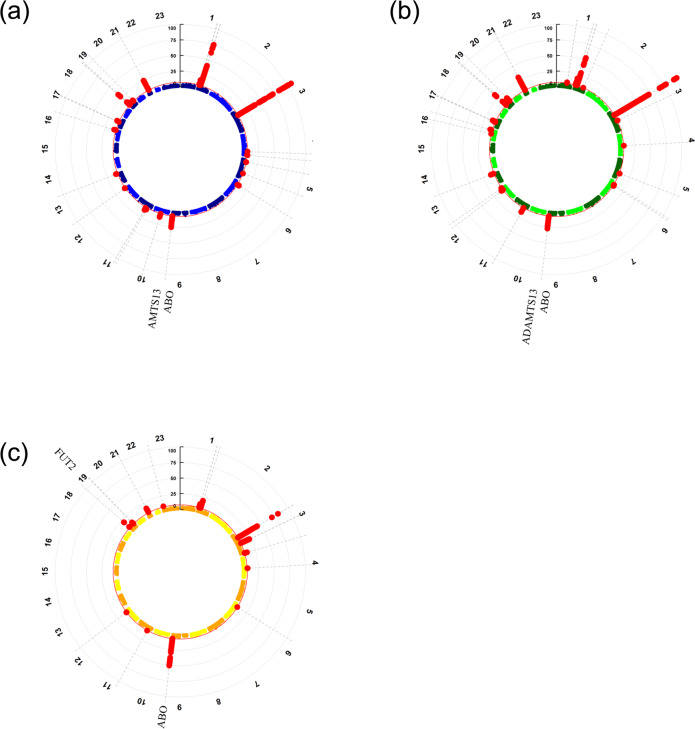
Circular Manhattan plot of cross-trait analysis. **a** VTE and severe COVID-19; **b** VTE and COVID-19 hospitalization; **c** VTE and SARS-CoV-2 infection. Each point represents a SNP, and significant loci with meta-analysis *P* value <5 × 10^−8^ are colored in red. Shared loci with meta-analysis *P* value < 5 × 10^−8^ and single-trait *P* value <5 × 10^−3^ are labeled with the mapped genes. SNPs are arranged according to the chromosome position. The presented data are available in Supplementary Data 8–10.

As shown in Table 1, we identified 2 genetic loci associated with both VTE and severe COVID-19, 3 with both VTE and COVID-19 hospitalization, and 4 with both VTE and SARS-CoV-2 infection (*P*_meta_ < 5 × 10^−8^; single trait *P* < 5 × 10^−3^). In line with previous studies^3–6,14,15^, we identified *ABO* gene and *FUT2* gene, which contributed to both VTE and COVID-19. The strongest association signals were localized on or near the *ABO* gene (index SNP: rs11244061 for severe COVID-19 and COVID-19 hospitalization; rs550057 for SARS-CoV-2 infection) at locus 9q34.2. We also identified *ADAMTS13*, which has not been reported yet. Figure 3 displays the Manhattan plots of these results (also shown in Supplementary Data 8–10).

**Table 1:** Genome-wide significant loci associated with the COVID-19 traits and VTE in cross-trait meta-analysis.

SNP	CHR	A1	A2	Representative gene	BETA_1	SE_1	P_1	BETA_2	SE_2	P_2	P_META	Conditional posterior probability of colocalization (P(H_4_)/(P(H_3_) + P(H_4_)
Severe COVID-19 and VTE												
rs11244061	9	T	C	near ABO	0.9528	0.3582	1.36E + 08	0.1145	0.1076	1.33E + 08	3.55E-26	8.55E-01
rs149181677	9	T	C	ADAMTS13	0.9785	0.3064	1.36E + 08	0.0526	0.1192	1.33E + 08	2.07E-10	8.55E-01
COVID-19 hospitalization and VTE												
rs11244061	9	T	C	near ABO	0.9528	0.3582	1.36E + 08	0.1070	0.0994	1.33E + 08	3.64E-28	9.00E-01
rs149181677	9	T	C	ADAMTS13	0.9785	0.3064	1.36E + 08	0.0530	0.0978	1.33E + 08	1.09E-10	9.00E-01
rs8176686	9	C	C	ABO	0.6813	−0.1300	1.36E + 08	0.4499	−0.0709	1.33E + 08	1.89E-14	9.00E-01
SARS-CoV-2 infection and VTE												
rs492602	19	G	A	FUT2	0.6754	0.0927	4.92E + 07	0.4707	−0.0357	4.87E + 07	1.42E-10	8.23E-01
rs550057	9	T	C	ABO	0.7830	0.3176	1.36E + 08	0.7402	−0.0963	1.33E + 08	1.56E-51	9.84E-01
rs71503180	9	A	G	near ABO	0.9757	−0.1391	1.36E + 08	0.0693	−0.0506	1.33E + 08	3.05E-10	9.84E-01
rs9411367	9	T	C	near ABO	0.6740	0.1155	1.36E + 08	0.1948	0.0380	1.33E + 08	3.20E-12	9.84E-01

*CHR* chromosome, *A1* other allele, *A2* effect allele.

BETA_1, SE_1, and P_1 represent the estimated effects, standard errors, and *p* values in the GWAS of VTE. BETA_2, SE_2, and P_2 represent the estimated effects, standard errors and, *p* values in the GWASs of COVID-19-related traits. P_META represents the *p* value of the cross-trait meta-analysis.

### Fine-mapping and colocalization analysis identify shared causal variants

For each loci associated with VTE and COVID-19, Supplementary Tables 3–5 listed all SNPs within 500 kb of these variants in the 99% credible sets. Co-localization analysis shows that all loci identified in cross-trait meta-analysis had the conditional posterior probability of colocalization >0.8, (Table 1), indicating that they are shared loci between the two traits.

### GTEx tissue-specific expression analysis (TSEA) and over-representation enrichment analysis of shared genes

**Fig. 4:**
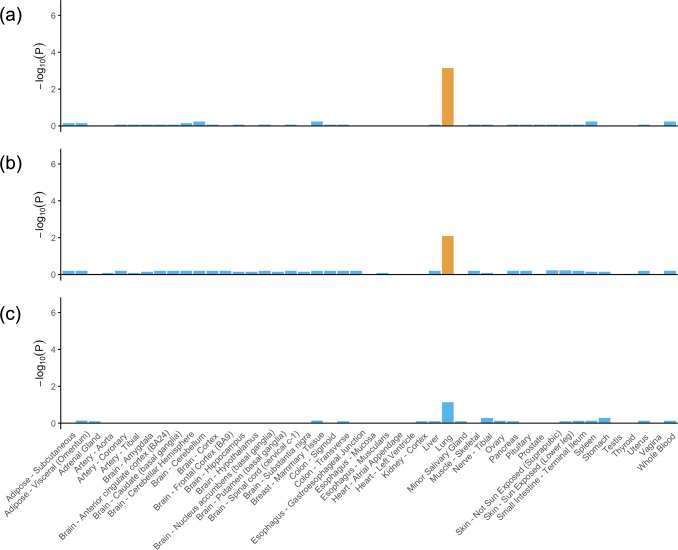
GTEx tissue enrichment analysis for expression of shared significant genes (meta-analysis *P* value < 5 × 10^−6^). **a** VTE and severe COVID-19; **b** VTE and COVID-19 hospitalization; **c** VTE and SARS-CoV-2 infection. *P* values of Fisher’s exact test after Benjamin−Hochberg correction are presented in –log_10_ scale. Orange represents significant tissue enrichment (Lung, *P* value = 7.16 × 10^−4^ for severe COVID-19 and VTE, *P* value = 8.20 × 10^−3^ for COVID-19 hospitalization and VTE). The presented data are available in Supplementary Data 11.

The potential associated genes identified for severe COVID-19 and COVID-19 hospitalization with VTE were significantly enriched for expression in the lung tissue; however, no highly enriched tissues were found for SARS-CoV-2 infection and VTE (Fig. 4 and Supplementary data 11). Gene ontology (GO) analysis highlighted several significant shared biological processes between three COVID-19 traits and VTE, such as “calcium-mediated signaling”, “second-messenger-mediated signaling”, “chemokine-mediated signaling pathway”, “response to chemokine”, “response to type I interferon” (Supplementary Tables 6–8).

### Bi-directional MR analysis

**Fig. 5:**
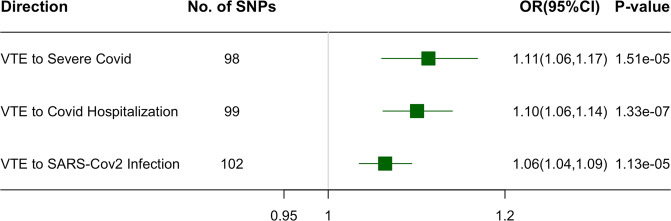
Mendelian randomization analysis on the association of genetically predicted VTE with the risk of COVID-19-related traits using inverse variance weighting (IVW). The estimates are presented as odds ratios (OR) with 95% confidence intervals (CI). The presented data are available in Supplementary Data 12.

**Fig. 6:**
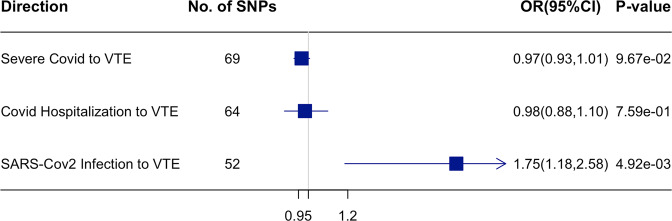
Mendelian randomization analysis on the association of genetically predicted COVID-19-related traits with the risk of VTE using IVW. The estimates are presented as odds ratios (OR) with 95% confidence intervals (CI). An arrow represents the estimate that is out of the boundary in this direction. The presented data are available in Supplementary Data 12.

We found that genetically predicted VTE was positively associated with higher risk of all three COVID-19-related traits (OR = 1.11 for severe COVID-19, 95% CI: 1.06–1.17, *P* value = 1.51 × 10^-5^; OR = 1.10 for COVID-19 hospitalization, 95% CI: 1.06–1.14, *P* value = 1.33 × 10^−^^7^; OR = 1.06 for SARS-CoV-2 infection, 95% CI: 1.04–1.09, *P* value = 1.13 × 10^−5^) (Fig. 5 and Supplementary Data 12). MR-Egger intercept test did not indicate directional pleiotropy (Supplementary Table 9), which supported the validity of the findings. MR-PRESSO detected outliers in the associations of VTE with three COVID-19 traits (shown in Supplemental Data 3), and the positive associations of VTE with severe COVID-19 and COVID-19 hospitalization remained after removing these outliers (Supplementary Figure 1). In the reverse direction, we found a positive association of genetically predicted SARS-CoV-2 infection with higher risk of VTE (OR = 1.75, 95% CI: 1.18–2.58, *P* value = 4.92 × 10^−3^), and null associations of genetically predicted severe COVID-19 or COVID-19 hospitalization with VTE (Fig. 6 and Supplementary Data 12). These results are robust to different MR methods (Supplementary Figure 2). And we obtained similar bi-directional associations between VTE and COVID-19 if we used COVID-19 HGI release 7 excluding data from UK Biobank (Supplementary Table 10).

## Discussion

In this study, we comprehensively investigated the shared genetic etiology between three COVID-19 related traits and VTE based on the latest data from COVID-19 HGI and UK Biobank. We found a significant positive genetic correlation of VTE with COVID-19 hospitalization and identified specific shared loci for VTE with each COVID-19 trait in *ABO, ADAMTS13* and *FUT2* genes, which was involved in coagulation. Enrichment analysis suggested that genes potentially associated with VTE, and COVID-19 were enriched for expression in the lung tissue, and supported pathways related to coagulation and immunity. Moreover, we examined their causal relationship using bi-directional MR, which suggested that VTE may increase the risk of severe COVID-19, COVID-19 hospitalization, and SARS-CoV-2 infection, and interestingly, SARS-CoV-2 infection may increase the risk of VTE.

LDSC analysis in our study found genetic correlation between VTE and COVID-19, which provided genetic evidence of a significant positive genetic correlation between VTE and COVID-19 hospitalization. Although LDSC analysis did not find significant overall genetic correlation between severe COVID-19 and VTE, however partitioned LDSC analysis found severe COVID-19 and VTE was positively correlated in some functional categories, including TFBS, Fetal DHS, H3K4me1, H3K9ac and H3K27ac, which are associated with the control of transcription and the status of *cis*-regulatory elements such as promoters and enhancers within gene regulatory regions^16–19^.

Notably, we identified several shared genetic loci between VTE and COVID-19 using cross-trait meta-analysis and colocalization analysis. These loci were located on or near the *ABO*, *FUT2*, and *ADAMST13* genes, which are well-established VTE related genes^20,21^. Our findings are consistent with previous genetic studies showing *ABO* gene is associated with severe COVID-19 and SARS-CoV-2 infection^3–5,14^, possibly by regulating thrombosis^3,22^, as *ABO* gene is responsible for post-translational glycosylation of coagulation factors. In line with the role of *ABO* gene, we found that *FUT2* gene, a fucosyltransferase gene involved in ABO blood group antigen synthesis, was associated with both VTE and SARS-CoV-2 infection. We also found a shared gene, *ADAMST13*, encoding the protein ADAMST13, which has not been previously reported in COVID-19 GWAS. Defects in this gene are associated with blood clotting and thrombotic thrombocytopenic purpura^23^, and a previous MR study showed genetically predicted ADAMST13 level is associated with severe COVID-19^24^.

In addition, findings from enrichment analysis suggest immune function might be involved in the shared etiology. In the enrichment analysis, we found several shared pathways related to immune function, such as calcium-mediated signaling and chemokine/interferon related response. Previous studies suggested that calcium signaling may play a role in hemostasis and thrombosis^25,26^. Calcium signaling is also of paramount importance in immune cells^27,28^, and chemokine and interferon play a major role in activating host immune and inflammatory responses^29–31^. Our TSEA reported that shared genes for severe COVID-19 and COVID-19 hospitalization with VTE were mainly enriched for gene expressions in the lung tissue. Consistently, pulmonary vascular endothelial injury and immunothrombosis (most of which occur within the lung microvessels) are key drivers of severe events after SARS-CoV-2 infection, such as acute respiratory failure and ARDS^32,33^. So, these evidence suggests that the common pathways between COVID-19 and VTE may relate to immunity, endothelial cell function, and coagulation.

Our study also provided timely evidence regarding the causal relationship between VTE and COVID-19 traits. Interestingly, our MR analysis showed a positive association between genetic susceptibility for VTE and the risk of severe COVID-19, COVID-19 hospitalization and SARS-CoV-2 infection, as well as a positive association of SARS-CoV-2 infection with VTE. Despite the statistical significance, our finding still needs to be interpreted with caution as we cannot exclude that this positive association may have occurred by chance. However, the positive association was also shown in a previous MR study using COVID-19 HGI release 5 data which showed that genetically predicted VTE was associated with higher risk of COVID-19 hospitalization and SARS-CoV-2 infection^13^. Using COVID-19 HGI release 7 with doubled sample size of release 5, we added by showing a positive association of genetically predicted VTE with higher risk of severe COVID-19. In addition, there are concerns that partial overlap of the samples may bias the estimates, however, we obtained consistent associations when we used COVID-19 HGI release 7 excluding data from UK Biobank. We also considered that the observed associations may be mediated by coagulation factors, as VTE is associated with coagulation factors^34^, and coagulation is a risk factor of COVID-19^35,36^. Although a previous MR study on 12 coagulation factors and COVID-19 severity found that VWF was link to COVID-19 severity using COVID-19 HGI release 5^24^, we found null associations of these coagulation factors with three COVID-19 traits using the latest and much larger GWAS of COVID-19, COVID-19 HGI release 7 (Supplementary Figure 3). Further studies are needed to explore whether there are other potential mediators.

Our study delivers an important information that VTE possibly increase the risk of COVID-19, which matters in the consideration of COVID-19 management strategies, especially for people with VTE. For example, taking vaccination to lower the risk for severe COVID-19 in people with VTE. Meanwhile, our MR analysis suggested that genetically predicted SARS-CoV-2 infection was possibly associated with a higher risk of VTE. Partly consistent with our results, in a cohort of 153,760 individuals with COVID-19, as well as two sets of control cohorts with 5,637,647 (contemporary controls) and 5,859,411 (historical controls) individuals, people with SARS-CoV-2 infection had higher risk of cardiovascular events, including VTE^37^. Nevertheless, a replication study of MR analysis in a larger GWAS of VTE will be worthwhile.

To the best of our knowledge, our study is the first to use large-scale genetic data to explore the shared genetic architecture between COVID-19 related traits and VTE, providing timely evidence and more insights into the genetic etiology between them. The GWAS summary-level data for COVID-19 was obtained from the COVID19 HGI release 7 summary statistics, the largest and latest GWAS of COVID-19 available, which doubles the sample size from the previous version, and improves study power.

We also acknowledge several limitations of our study. First, the GWAS data for COVID-19 and VTE used in this study were derived from the European population, so the associations may not be generalizable to other ancestries. Second, although GWAS summary statistics conducted study-specific quality control, misclassification of COVID-19 might exist. Third, the summary statistics limit us to assess sex and age-specific genetic effects. Fourth, the GWAS of COVID-19 related traits might be conducted at different time periods, so there might be differences in the SARS-CoV-2 infection strain. However, our study does not aim to assess the association with specific strain of SARS-CoV-2 infection. Fifth, genetic instruments for COVID-19 only capture a small proportion of variance. Although MR analysis provided unconfounded estimates, it is less precise than conventional observational studies. We observed a positive association of genetically predicted SARS-CoV-2 infection with VTE using inverse variance weighting and MR-RAPS, but the association included the null in weighted median, MR-Egger, MR-PRESSO and weighted mode, which may be due to the wider confidence intervals. Replication in a larger GWAS will be needed for validation. Finally, although our study provides evidence of genetic correlation and genetic overlap between COVID-19 and VTE, the underlying biological mechanisms are still unclear. For example, we identified rs149181677, the variant located on the *ADAMTS13* gene, was shared between COVID-19 and VTE. Although this SNP was associated with the expression of *ADAMTS13* genes^38^, we cannot fully confirm the link between this genetic variant with the protein ADAMTS13, as there is no study assessing the association between this genetic variant with ADAMTS13. Future studies are warranted to test the link.

In conclusion, our findings provided important evidence of genetic correlations between severe COVID-19, COVID-19 hospitalization, SARS-CoV-2 infection and VTE, and highlighted their common genetic architecture, with shared genes closely related to coagulation and immunity. We also found a causal association of VTE with COVID-19. Our work contributes to the understanding of COVID-19 and VTE etiology and provides more insights into the prevention and comorbidity management of COVID-19.

## Methods

### Study population

The GWAS summary statistics for COVID‐19 of European ancestry were provided by the COVID-19 host genetics initiative (COVID-19 HGI) round 7 (https://www.covid19hg.org/results/, release date: 08 April 2022). COVID-19 HGI is the largest GWAS of COVID-19 to date, combining data from over 3 million individual samples across 82 large cohort studies in 35 countries. We included three COVID-19 related traits: (1) Severe COVID-19, defined as COVID-19-confirmed individuals with very severe respiratory symptoms or those who died from the disease (up to 13,769 cases and 1,072,442 controls); (2) COVID-19 hospitalization defined as individuals who were hospitalized for related infection symptoms, with laboratory-confirmed SARS-CoV-2 infection (up to 32,519 cases and 2,062,805 controls); (3) SARS-CoV-2 infection defined as all individuals who reported positive (laboratory diagnosis, physician diagnosis or self-report) for SARS-CoV-2 infection (up to 122,616 cases and 2,475,240 controls). For VTE, we used summary statistics on the GWAS of VTE (3900 cases and 369,592 controls of European ancestry) from the UK Biobank provided by Lee Lab (https://www.leelabsg.org/resources).

### Ethics approval

Participants for all studies included in COVID-19 HGI were recruited following protocols approved by the local institutional review boards and informed consent was obtained where required^4^. The UK Biobank has already received the ethical approval from North West Multi-center Research Ethics Committee (MREC), which covers the UK. It also got the approval from the Patient Information Advisory Group (PIAG) in England and Wales and from the Community Health Index Advisory Group (CHIAG) in Scotland. This study is an analysis using publicly available summary data that does not require additional ethical approval.

### Linkage disequilibrium score regression (LDSC) analysis

LDSC analysis was conducted to assess the heritability for a single trait and genome-wide genetic correlations between two traits, where genome-wide associations were used in the calculation. The analysis was conducted using the LDSC software based on the GWAS summary statistics. LD scores of 1000 G European ancestry was used as reference^39^

An estimate of the heritability or genetic correlation was obtained by regressing the *χ*^2^ statistics or the products of *z* scores on LD scores, respectively. LDSC can also correct for the inflation of test statistics caused by polygeneicity^40^. Under the polygenic model, the expected ${{{{{{\rm{\chi }}}}}}}^{2}$ statistic and the LD score ${{{{{{\rm{l}}}}}}}_{{{{{{\rm{j}}}}}}}$ of SNP ${{{{{\rm{j}}}}}}$ follows the linear relationship:^39^

1 $${{{{{\rm{E}}}}}}\left[{{{{{{\rm{\chi }}}}}}}_{j}^{2} | {{{{{{\rm{l}}}}}}}_{{{{{{\rm{j}}}}}}}\right]=\frac{{{{{{\rm{N}}}}}}{{{{{{\rm{h}}}}}}}^{2}{l}_{j}}{{{{{{\rm{M}}}}}}}+{Na}+1$$

where ${{{{{\rm{N}}}}}}$ is the sample size, ${{{{{\rm{M}}}}}}$ is the number of SNPs, ${{{{{{\rm{h}}}}}}}^{2}$ is the heritability, and ${{{{{\rm{a}}}}}}$ measures the cofounding bias. Therefore, an estimate of the heritability can be obtained by regressing the ${{{{{{\rm{\chi }}}}}}}^{2}$ statistics calculated from GWAS on the LD scores from a reference panel.

If we have two studies for two polygenic traits, there is a similar relationship between the product of the ${{{{{\rm{z}}}}}}$-scores from two studies and the LD scores:^41^

2 $${{{{{\rm{E}}}}}}\left[{{{{{{\rm{z}}}}}}}_{1{{{{{\rm{j}}}}}}}{{{{{{\rm{z}}}}}}}_{2{{{{{\rm{j}}}}}}} | {{{{{{\rm{l}}}}}}}_{{{{{{\rm{j}}}}}}}\right]=\frac{\sqrt{{{{{{{\rm{N}}}}}}}_{1}{N}_{2}}{\rho }_{g}}{M}{{{{{{\rm{l}}}}}}}_{{{{{{\rm{j}}}}}}}+\frac{\rho {N}_{s}}{\sqrt{{{{{{{\rm{N}}}}}}}_{1}{N}_{2}}}$$

where ${{{{{{\rm{N}}}}}}}_{{{{{{\rm{i}}}}}}}$ is the sample size for study ${{{{{\rm{i}}}}}}$, ${\rho }_{g}$ is the genetic covariance, ${N}_{s}$ is the number of overlapping samples, and $\rho$ is the phenotypic correlation. If we regress the product of the ${{{{{\rm{z}}}}}}$-scores from two GWASs on the LD scores from a reference panel, we can get an estimate of the genetic covariance between two traits. The genetic correlation then can be obtained by ${{{{{{\rm{r}}}}}}}_{{{{{{\rm{g}}}}}}}={\rho }_{g}/\sqrt{{h}_{1}^{2}{h}_{2}^{2}}$, where ${{{{{{\rm{h}}}}}}}_{{{{{{\rm{i}}}}}}}^{2}$ is the heritability of trait ${{{{{\rm{i}}}}}}$.

### Partitioned LDSC analysis

We performed partitioned LDSC analysis to estimate the genetic correlation between two traits within each of the following 12 functional categories:^17^ conserved region, DNaseI digital genomic footprinting region (DGF), DNase I hypersensitivity sites (DHS), fetal DHS, H3K4me1, H3K4me3, H3K9ac and H3K27ac, intron region, super enhancers, transcription factor-binding site (TFBS) and transcribed region. The re-calculated LD scores of the SNPs classified in each specific annotation category allowed us to find out which functional categories accounted for the majority of the overall genetic correlation.

### Multi-trait analysis of GWAS

We applied MTAG, an approach for meta-analysis of summary statistics from GWAS of different traits robust to sample overlap, to identify novel loci with strong signals for COVID-19, and to detect genome-wide significant loci between VTE and three COVID-19 traits^42–44^.

MTAG can improve the effect estimates for each COVID-19 trait by incorporating the weighted sum of GWAS estimates for VTE. Let ${{{{{{\boldsymbol{\beta }}}}}}}_{{{{{{\rm{j}}}}}}}$ be the true effects of SNP ${{{{{\rm{j}}}}}}$ on multiple traits with ${{{{{\rm{E}}}}}}\left[{{{{{{\boldsymbol{\beta }}}}}}}_{{{{{{\rm{j}}}}}}}\right]={{{{{\bf{0}}}}}}$ and ${{{{{\rm{Var}}}}}}\left({{{{{{\boldsymbol{\beta }}}}}}}_{{{{{{\rm{j}}}}}}}\right)={{{{{\boldsymbol{\Omega }}}}}}$. MTAG assumes that ${{{{{\boldsymbol{\Omega }}}}}}$ is the same for all SNPs. Based on the moment condition, the MTAG estimates can be expressed as a weighted sum of the GWAS estimates:

3 $${\hat{\beta }}_{{MTAG},j,t}=\frac{\frac{{{{{{{\boldsymbol{\omega }}}}}}}_{{{{{{\boldsymbol{t}}}}}}}{{{\prime} }}}{{\omega }_{{tt}}}{\left({{{{{\boldsymbol{\Omega }}}}}}{{{{{\boldsymbol{-}}}}}}\frac{{{{{{{\boldsymbol{\omega }}}}}}}_{{{{{{\boldsymbol{t}}}}}}}{{{{{{\boldsymbol{\omega }}}}}}}_{{{{{{\boldsymbol{t}}}}}}}{{{\prime} }}}{{\omega }_{{tt}}}+{{{{{{\boldsymbol{\Sigma }}}}}}}_{{{{{{\boldsymbol{j}}}}}}}\right)}^{-1}}{\frac{{{{{{{\boldsymbol{\omega }}}}}}}_{{{{{{\boldsymbol{t}}}}}}}{{{\prime} }}}{{\omega }_{{tt}}}{\left({{{{{\boldsymbol{\Omega }}}}}}{{{{{\boldsymbol{-}}}}}}\frac{{{{{{{\boldsymbol{\omega }}}}}}}_{{{{{{\boldsymbol{t}}}}}}}{{{{{{\boldsymbol{\omega }}}}}}}_{{{{{{\boldsymbol{t}}}}}}}{{{\prime} }}}{{\omega }_{{tt}}}+{{{{{{\boldsymbol{\Sigma }}}}}}}_{{{{{{\boldsymbol{j}}}}}}}\right)}^{-1}\frac{{{{{{{\boldsymbol{\omega }}}}}}}_{{{{{{\boldsymbol{t}}}}}}}}{{\omega }_{{tt}}}}{\hat{{{{{{\boldsymbol{\beta }}}}}}}}_{{{{{{\boldsymbol{j}}}}}}}$$

where ${{{{{{\boldsymbol{\omega }}}}}}}_{{{{{{\boldsymbol{t}}}}}}}$ is the ${{{{{\rm{t}}}}}}$-th column of ${{{{{\boldsymbol{\Omega }}}}}}$, ${\omega }_{{tt}}$ is the ${{{{{\rm{t}}}}}}$-th diagonal entry of ${{{{{\boldsymbol{\Omega }}}}}}$, ${{{{{{\boldsymbol{\Sigma }}}}}}}_{{{{{{\boldsymbol{j}}}}}}}$ is the covariance of the GWAS estimates ${\hat{{{{{{\boldsymbol{\beta }}}}}}}}_{{{{{{\boldsymbol{j}}}}}}}$. Therefore, we can check whether there are novel loci that are extracted from the MTAG estimates and not identified using GWAS.

We also used MTAG to conduct genome-wide cross-trait meta-analysis, which utilizes sample size-weighted, fixed-effect model together with genetic covariance modeling from all sources to combine evidence of the genome-wide association between individual variants for VTE and COVID-19. The equation for the MTAG estimates can be simplified to.:

4 $${\hat{\beta }}_{{MTAG},j,t}=\frac{{{{{{{{\bf{1}}}}}}}^{{\prime} }{{{{{\boldsymbol{\Sigma }}}}}}}_{{{{{{\boldsymbol{j}}}}}}}^{-{{{{{\bf{1}}}}}}}\,}{{{{{{{{\bf{1}}}}}}}^{{\prime} }{{{{{\boldsymbol{\Sigma }}}}}}}_{{{{{{\boldsymbol{j}}}}}}}^{-{{{{{\bf{1}}}}}}}{{{{{\bf{1}}}}}}}{\hat{{{{{{\boldsymbol{\beta }}}}}}}}$$

Under this assumption, MTAG can summarize the effect estimates from two GWASs and produce cross-trait effect estimates, which allows us to detect the shared significant loci between two traits.

Using cross-trait meta-analysis, we identified SNPs reaching genome-wide significance (*P*_meta_ < 5 × 10^−8^) in both traits and suggestive significance (single trait *P* < 5 × 10^−3^) in single traits^45^. We further applied the PLINK clumping function (*r*^2^ threshold = 0.01, distance = 500 kb) to ensure the independence of selected SNPs, i.e., SNPs in linkage disequilibrium that have a correlation >0.01 with the most significant SNP within a distance of 500 kb will be pruned^46^.

### Fine-mapping and co-localization analysis

For each locus associated with two traits, we used the Bayesian fine-mapping algorithm that assumes a multinomial likelihood for the joint distribution of the phenotypes and genotypes to identify a 99%-credible set of causal variants within 500 kb of the index SNP^47,48^. This algorithm uses a flat prior with steepest descent approximation and only requires summary statistics. Then, we conducted the co-localization analysis to check whether the loci identified in the cross-trait meta-analysis are causal variants shared between COVID-19 and VTE. The SNP causality between two traits in a region can be assigned to one of the following five hypotheses^49^:

${H}_{0}:$ No association with either trait.

${H}_{1}:$ Association with trait 1 only.

${H}_{2}:$ Association with trait 2 only.

${H}_{3}:$ Association with both traits, two independent SNPs.

${H}_{4}:$ Association with both traits, one shared SNP.

With the R package ‘coloc’, we can calculate the posterior probability for each hypothesis based on the summary statistics. In colocalization, we calculated the conditional posterior probability of colocalization (P(H_4_)/(P(H_3_) + P(H_4_))^49^. If a SNP has the probability larger than 0.8, we labeled it as a co-localized genetic variant.

### TSEA and over-representation enrichment analysis

We also performed TSEA with the HUGO Gene Nomenclature Committee (HGNC) name on genes that correspond with the associated loci with meta-analysis *P* value < 5 × 10^-6^ in cross-trait meta-analysis to test whether these genes are overly expressed in a specific tissue. We used the R package ‘deTS’, which uses GTEx RNA-seq data and the ENCODE panel as a reference panel, and calculates the corresponding *z* score for each tissue^50^. We also used the WEB-based GEne SeT AnaLysis Toolkit to further assess the overrepresented enrichment of the same genes in Gene ontology (GO) biological process^51^.

### Mendelian randomization (MR) analysis

We conducted a bidirectional MR analysis to assess the causal association between each COVID-19 related trait and VTE. Genetic variants for VTE were obtained from a large GWAS of VTE restricted to Europeans (23,151 cases, 553,439 controls)^52^. We extracted genome-wide significant (*P* < 5 × 10^−8^) and uncorrelated (*r*^2^ < 0. 2) SNPs, and then excluded six SNPs in *ABO, FUT2, VWF* genes (Supplemental Data 3) as these gene regions have pleiotropic associations with multiple traits, including COVID-19. 107 SNPs has been selected as genetic instruments for VTE (Supplementary Data 3)^52^. The genetic associations of these SNPs with COVID-19-related traits were obtained from COVID-19 HGI release 7 GWAS meta-analysis, as shown in study population. Using the same process of identifying instruments (*P* < 5 × 10^−8^, *r*^2^ < 0. 2), and after excluding 2, 2, and 8 SNPs in *ABO, FUT2,* or *VWF* genes, we obtained 87, 83, and 61 SNPs as instruments for severe COVID-19, COVID-19 hospitalization and SARS-CoV-2 infection respectively based on COVID-19 HGI release 7 GWAS meta-analysis (Supplementary Data 4–6). The genetic associations of these SNPs with VTE were obtained from the GWAS of VTE (3900 cases and 369,592 controls of European ancestry) in the UK Biobank. We used inverse variance weighted (IVW) analysis in the main analysis^53^, and used several other MR methods that are robust to pleiotropy, including the weighted median^54^, MR-Egger^55^, MR-PRESSO^56^, MR-RAPS^57^, and weighted mode^58^ in sensitivity analysis. Considering sample overlap, we also repeated the bi-directional analysis in the sensitivity analysis using the GWAS summary statistics for COVID‐19 excluding data from UK Biobank provided by COVID-19 HGI release 7 (https://www.covid19hg.org/results/).

### Statistics and reproducibility

We used the following software and packages: R and R packages: R (version 4.0.5), TwoSampleMR (version 0.5.6), forestplot (version 2.0.1), mr.raps (version 0.2), dplyr (version 1.0.5), CMplot (version 3.6.2), corrplot (version 0.92), ggplot2 (version3.3.5), deTS (version 1.0), WebGestaltR (version 0.4.4), coloc (version 3.2-1), data.table (version 1.14.2), gprofiler2 (version 0.2.1). Python and Python-based software: Python (version 2.7.5 and 3.6.3), LDSC (version 1.0.1), MTAG (version 1.0.8). We used publicly available GWAS data with up to 13,769 cases and 1,072,442 controls for severe COVID-19; up to 32,519 cases and 2,062,805 controls for COVID-19 hospitalization; up to 122,616 cases and 2,475,240 controls for SARS-CoV-2 infection. To enable us to test the reproducibility of causal associations between VTE and three COVID-19 traits obtained from MR analysis, we used COVID-19 data excluding the UK Biobank for further analysis and obtained similar results (Supplementary Table 10).

### Reporting summary

Further information on research design is available in the Nature Portfolio Reporting Summary linked to this article.

## Supplementary information

Peer Review File

Supplementary Information

Description of Additional Supplementary Files

Supplementary Data 1–12

Reporting Summary

## Data Availability

Genetic associations with VTE were from the UK biobank GWAS results provided by Lee Lab (https://www.leelabsg.org/resources). Genetic associations with severe COVID-19, COVID-19 hospitalization and SARS-CoV-2 infection were obtained from COVID-19 host genetics consortium GWAS meta-analyses round 7, downloaded from https://storage.googleapis.com/covid19-hg-public/freeze_7/results/20220403/pop_spec/sumstats/COVID19_HGI_A2_ALL_eur_leave23andme_20220403.tsv.gz; https://storage.googleapis.com/covid19-hg-public/freeze_7/results/20220403/pop_spec/sumstats/COVID19_HGI_B2_ALL_eur_leave23andme_20220403.tsv.gz; https://storage.googleapis.com/covid19-hg-public/freeze_7/results/20220403/pop_spec/sumstats/COVID19_HGI_C2_ALL_eur_leave23andme_20220403.tsv.gz. In the MR sensitivity analysis, Genetic associations with severe COVID-19, COVID-19 hospitalization and SARS-CoV-2 infection excluding data from UK Biobank were obtained from https://storage.googleapis.com/covid19-hg-public/freeze_7/results/20220403/leave_one_out/sumstats/COVID19_HGI_A2_ALL_leave_23andme_and_UKBB_20220403.tsv.gz; https://storage.googleapis.com/covid19-hg-public/freeze_7/results/20220403/leave_one_out/sumstats/COVID19_HGI_B2_ALL_leave_23andme_and_UKBB_20220403.tsv.gz; https://storage.googleapis.com/covid19-hg-public/freeze_7/results/20220403/leave_one_out/sumstats/COVID19_HGI_C2_ALL_leave_23andme_and_UKBB_20220403.tsv.gz. Source data underlying Figs. 2–6 are provided in Supplementary Data 7–12

## Code availability

We used publicly available software for analysis in this study. Here, we listed the URLs (some of which are online methods) for the software: TwoSampleMR, forestplot, mr.raps, deTS, WebGestaltR, coloc, gprofiler2, LDSC, MTAG. Detailed code book, and analytic code will be made available upon request pending application and approval by the corresponding author.
