# Supplementary material for: Genome-wide cross-trait analysis and Mendelian randomization reveal a shared genetic etiology and causality between COVID-19 and venous thromboembolism
Source: Commun Biol. 2023 Apr 21;6:441. doi: 10.1038/s42003-023-04805-2 (PMC10120502; doi:10.1038/s42003-023-04805-2)
Supplement: Supplementary file 2 — Supplementary Information [file 42003_2023_4805_MOESM2_ESM.pdf]

Supplementary Table 1. SNP based heritability estimated by LDSC

Phenotype	Heritability (h2)	Heritability SE	Heritability P
VTE	0.0094	0.0019	7.52E-07
Severe COVID-19	0.0064	0.0012	9.64E-08
COVID-19 hospitalization	0.0036	0.0006	1.97E-09
SARS-CoV-2 infection	0.0020	0.0003	2.62E-11

Supplementary Table 2. SNP based genetic correlation estimated by LDSC

Phenotype 1	Phenotype 2	Genetic Correlation	Genetic Correlation SE	Genetic Correlation P
VTE	Severe COVID-19	0.0573	0.0867	0.5087
	COVID-19 hospitalization	0.2320	0.0891	0.0092
	SARS-CoV-2 infection	0.1753	0.0978	0.0731

Supplementary Table 3. List of credible set SNPs in each locus from fine mapping. For each of the VTE and severe COVID-19 shared loci, the table lists all SNPs within 500 kb of these variants in the 99% credible sets that were calculated.

Sentinel SNP	Sentinel SNP in credible-set	99% credible-set SNPs	CHR	BP	VTE	Severe COVID-19	Cross trait meta-analysis
					cumSum	cumSum	cumSum
rs11244061	TRUE	rs11244061	9	136153981	NA	NA	0.999
rs149181677	TRUE	rs149181677	9	136296530	1	0.685	1

Supplementary Table 4. List of credible set SNPs in each locus from fine mapping. For each of the VTE and COVID-19 hospitalization shared loci, the table lists all SNPs within 500 kb of these variants in the 99% credible sets that were calculated.

Sentinel SNP	Sentinel SNP in credible-set	99% credible-set SNPs	CHR	BP	VTE	COVID-19 hospitalization	Cross trait meta-analysis
					cumSum	cumSum	cumSum
rs11244061	TRUE	rs11244061	9	136153981	NA	NA	1
rs149181677	TRUE	rs149181677	9	136296530	1	0.508	1

Supplementary Table 5. List of credible set SNPs in each locus from fine mapping. For each of the VTE and SARS-CoV-2 infection shared loci, the table lists all SNPs within 500 kb of these variants in the 99% credible sets that were calculated.

Sentinel SNP	Sentinel SNP in credible-set	99% credible-set SNPs	CHR	BP	VTE	SARS-CoV-2 infection	Cross trait meta-analysis
					cumSum	cumSum	cumSum
rs550057	TRUE	rs643434	9	136142355	NA	0.988	0.696
		rs545971	9	136143372	0.532	0.946	0.983
		rs612169	9	136143442	0.922	0.807	0.913
		rs8176663	9	136144427	0.489	0.396	0.761
		rs491626	9	136144873	0.617	0.621	0.871
		rs494242	9	136145118	NA	0.86	0.536
		rs495203	9	136145240	0.702	0.509	0.821
		rs582118	9	136145471	0.404	0.693	0.952
		rs550057	9	136146597	NA	NA	0.415
		rs554833	9	136147160	0.898	0.264	0.63
		rs529565	9	136149500	0.262	0.969	0.996
rs71503180	TRUE	rs34764475	9	136061515	0.997	0.952	0.985
		rs77236853	9	136101633	NA	0.652	0.996
		rs71503180	9	136106974	0.772	1	0.535
rs9411367	FALSE	rs13299342	9	136141504	NA	1	1
		rs8176632	9	136152547	NA	0.874	0.889
rs492602	TRUE	rs35106244	19	49203829	0.736	0.995	0.989
		rs516246	19	49206172	0.875	0.98	0.973
		rs492602	19	49206417	0.869	0.643	0.31
		rs681343	19	49206462	0.858	0.78	0.561
		rs281377	19	49206603	NA	NA	0.983
		rs601338	19	49206674	0.863	0.882	0.768

	rs35866622	19	49218060	0.371	NA	0.992

Supplementary Table 6. GO biological process pathway analysis for VTE and severe COVID-19 (FDR < 0.05).

GO biological process complete	Link	raw P value	FDR.	Overlap genes
GO:0070098 (chemokine-mediated signaling pathway)	http://amigo.geneontology.org/amigo/term/GO:0070098	4.41E-10	1.92E-06	CCR3;XCR1;CCRL2;CCR1;CCR9;CCR5;CCR2;CXCR6
GO:1990868 (response to chemokine)	http://amigo.geneontology.org/amigo/term/GO:1990868	9.15E-10	1.92E-06	CCR3;XCR1;CCRL2;CCR1;CCR9;CCR5;CCR2;CXCR6
GO:1990869 (cellular response to chemokine)	http://amigo.geneontology.org/amigo/term/GO:1990869	9.15E-10	1.92E-06	CCR3;XCR1;CCRL2;CCR1;CCR9;CCR5;CCR2;CXCR6
GO:0019722 (calcium-mediated signaling)	http://amigo.geneontology.org/amigo/term/GO:0019722	2.52E-09	3.98E-06	CCR3;ATP1B1;XCR1;SELE;CCRL2;CCR1;CCR9;CCR5;CCR2;CXCR6
GO:0007204 (positive regulation of cytosolic calcium ion concentration)	http://amigo.geneontology.org/amigo/term/GO:0007204	7.64E-07	9.63E-04	CCR3;XCR1;CCRL2;CCR1;CCR9;CCR5;CCR2;F2;CXCR6
GO:0019932 (second-messenger-mediated signaling)	http://amigo.geneontology.org/amigo/term/GO:0019932	1.83E-06	1.73E-03	CCR3;ATP1B1;XCR1;SELE;CCRL2;CCR1;CCR9;CCR5;CCR2;CXCR6
GO:0006874 (cellular calcium ion homeostasis)	http://amigo.geneontology.org/amigo/term/GO:0006874	2.07E-06	1.73E-03	CCR3;ATP1B1;XCR1;CCRL2;CCR1;CCR9;CCR5;CCR2;F2;CXCR6
GO:0051480 (regulation of cytosolic calcium ion concentration)	http://amigo.geneontology.org/amigo/term/GO:0051480	2.20E-06	1.73E-03	CCR3;XCR1;CCRL2;CCR1;CCR9;CCR5;CCR2;F2;CXCR6
GO:0055074 (calcium ion homeostasis)	http://amigo.geneontology.org/amigo/term/GO:0055074	2.74E-06	1.92E-03	CCR3;ATP1B1;XCR1;CCRL2;CCR1;CCR9;CCR5;CCR2;F2;CXCR6
GO:0072503 (cellular divalent inorganic cation homeostasis)	http://amigo.geneontology.org/amigo/term/GO:0072503	4.03E-06	2.54E-03	CCR3;ATP1B1;XCR1;CCRL2;CCR1;CCR9;CCR5;CCR2;F2;CXCR6
GO:0060326 (cell chemotaxis)	http://amigo.geneontology.org/amigo/term/GO:0060326	5.87E-06	3.11E-03	CCR3;XCR1;CCRL2;CCR1;CCR9;CCR5;CCR2;CXCR6
GO:0072507 (divalent inorganic cation homeostasis)	http://amigo.geneontology.org/amigo/term/GO:0072507	5.92E-06	3.11E-03	CCR3;ATP1B1;XCR1;CCRL2;CCR1;CCR9;CCR5;CCR2;F2;CXCR6

GO:0006968 (cellular defense response)	http://amigo.geneontology.org/amigo/term/GO:0006968	3.68E-05	1.79E-02	CCR3;CCR9;CCR5;CCR2
GO:0098780 (response to mitochondrial depolarisation)	http://amigo.geneontology.org/amigo/term/GO:0098780	4.15E-05	1.87E-02	CDC37;AMBRA1;GBA
GO:0002407 (dendritic cell chemotaxis)	http://amigo.geneontology.org/amigo/term/GO:0002407	7.30E-05	3.07E-02	CCR1;CCR5;CCR2
GO:0036336 (dendritic cell migration)	http://amigo.geneontology.org/amigo/term/GO:0036336	1.05E-04	4.13E-02	CCR1;CCR5;CCR2

Supplementary Table 7. GO biological process pathway analysis for VTE and COVID-19 hospitalization (FDR < 0.05).

GO biological process complete	Link	raw P value	FDR.	Overlap genes
GO:0019722 (calcium-mediated signaling)	http://amigo.geneontology.org/amigo/term/GO:0019722	2.89E-10	1.82E-06	CCR3;ATP1B1;MAPT;SELE;CCRL2;XCR1;CCR1;CCR9;CCR5;CCR2;CXCR6
GO:0070098 (chemokine-mediated signaling pathway)	http://amigo.geneontology.org/amigo/term/GO:0070098	7.74E-10	2.44E-06	CCR3;CCRL2;XCR1;CCR1;CCR9;CCR5;CCR2;CXCR6
GO:1990868 (response to chemokine)	http://amigo.geneontology.org/amigo/term/GO:1990868	1.60E-09	2.52E-06	CCR3;CCRL2;XCR1;CCR1;CCR9;CCR5;CCR2;CXCR6
GO:1990869 (cellular response to chemokine)	http://amigo.geneontology.org/amigo/term/GO:1990869	1.60E-09	2.52E-06	CCR3;CCRL2;XCR1;CCR1;CCR9;CCR5;CCR2;CXCR6
GO:0019932 (second-messenger-mediated signaling)	http://amigo.geneontology.org/amigo/term/GO:0019932	4.48E-08	5.65E-05	CCR3;ATP1B1;LINC02210-CRHR1;MAPT;SELE;CCRL2;XCR1;CCR1;CCR9;CCR5;CCR2;CXCR6
GO:0060337 (type I interferon signaling pathway)	http://amigo.geneontology.org/amigo/term/GO:0060337	6.09E-07	5.49E-04	IRF1;IFNAR2;OAS3;OAS1;CDC37;TYK2
GO:0071357 (cellular response to type I interferon)	http://amigo.geneontology.org/amigo/term/GO:0071357	6.09E-07	5.49E-04	IRF1;IFNAR2;OAS3;OAS1;CDC37;TYK2
GO:0034340 (response to type I interferon)	http://amigo.geneontology.org/amigo/term/GO:0034340	8.58E-07	6.77E-04	IRF1;IFNAR2;OAS3;OAS1;CDC37;TYK2
GO:0072503 (cellular divalent inorganic cation homeostasis)	http://amigo.geneontology.org/amigo/term/GO:0072503	7.60E-06	5.33E-03	CCR3;ATP1B1;CCRL2;XCR1;CCR1;SLC39A8;CCR9;CCR5;CCR2;CXCR6
GO:0060326 (cell chemotaxis)	http://amigo.geneontology.org/amigo/term/GO:0060326	9.86E-06	6.22E-03	CCR3;CCRL2;XCR1;CCR1;CCR9;CCR5;CCR2;CXCR6
GO:0072507 (divalent inorganic cation homeostasis)	http://amigo.geneontology.org/amigo/term/GO:0072507	1.11E-05	6.37E-03	CCR3;ATP1B1;CCRL2;XCR1;CCR1;SLC39A8;CCR9;CCR5;CCR2;CXCR6

GO:0007204 (positive regulation of cytosolic calcium ion concentration)	http://amigo.geneontology.org/amigo/term/GO:0007204	1.32E-05	6.96E-03	CCR3;CCRL2;XCR1;CCR1;CCR9;CCR5;CCR2;CXCR6
GO:0060333 (interferon-gamma-mediated signaling pathway)	http://amigo.geneontology.org/amigo/term/GO:0060333	1.54E-05	7.48E-03	IRF1;OAS3;OAS1;NR1H2;CDC37
GO:0006874 (cellular calcium ion homeostasis)	http://amigo.geneontology.org/amigo/term/GO:0006874	2.90E-05	1.31E-02	CCR3;ATP1B1;CCRL2;XCR1;CCR1;CCR9;CCR5;CCR2;CXCR6
GO:0051480 (regulation of cytosolic calcium ion concentration)	http://amigo.geneontology.org/amigo/term/GO:0051480	3.31E-05	1.39E-02	CCR3;CCRL2;XCR1;CCR1;CCR9;CCR5;CCR2;CXCR6
GO:0055074 (calcium ion homeostasis)	http://amigo.geneontology.org/amigo/term/GO:0055074	3.70E-05	1.43E-02	CCR3;ATP1B1;CCRL2;XCR1;CCR1;CCR9;CCR5;CCR2;CXCR6
GO:0071346 (cellular response to interferon-gamma)	http://amigo.geneontology.org/amigo/term/GO:0071346	3.85E-05	1.43E-02	IRF1;OAS3;OAS1;NR1H2;CDC37;ADAMTS13
GO:0006968 (cellular defense response)	http://amigo.geneontology.org/amigo/term/GO:0006968	4.82E-05	1.69E-02	CCR3;CCR9;CCR5;CCR2
GO:0034341 (response to interferon-gamma)	http://amigo.geneontology.org/amigo/term/GO:0034341	7.10E-05	2.36E-02	IRF1;OAS3;OAS1;NR1H2;CDC37;ADAMTS13
GO:0002407 (dendritic cell chemotaxis)	http://amigo.geneontology.org/amigo/term/GO:0002407	8.94E-05	2.82E-02	CCR1;CCR5;CCR2
GO:0036336 (dendritic cell migration)	http://amigo.geneontology.org/amigo/term/GO:0036336	1.28E-04	3.85E-02	CCR1;CCR5;CCR2

Supplementary Table 8. GO biological process pathway analysis for VTE and SARS-CoV-2 infection (FDR < 0.05).

GO biological process complete	Link	raw P value	FDR.	Overlap genes
GO:0070098 (chemokine-mediated signaling pathway)	http://amigo.geneontology.org/amigo/term/GO:0070098	2.79E-07	1.00E-03	CCR3;XCR1;CCR1;CCR9;CCR2;CXCR6
GO:1990868 (response to chemokine)	http://amigo.geneontology.org/amigo/term/GO:1990868	4.77E-07	1.00E-03	CCR3;XCR1;CCR1;CCR9;CCR2;CXCR6
GO:1990869 (cellular response to chemokine)	http://amigo.geneontology.org/amigo/term/GO:1990869	4.77E-07	1.00E-03	CCR3;XCR1;CCR1;CCR9;CCR2;CXCR6
GO:0019722 (calcium-mediated signaling)	http://amigo.geneontology.org/amigo/term/GO:0019722	4.88E-06	7.69E-03	CCR3;ATP1B1;XCR1;CCR1;CCR9;CCR2;CXCR6

Supplementary Table 9. MR-Egger Intercept and the Corresponding Heterogeneity Q Statistic of Exposure-Outcome Pair.

Exposure	Outcome	Intercept	P-value of Intercept	Q statistic	P-value of Q Statistic
VTE	Severe COVID-19	-0.003	6.61E-01	171.480	3.49E-06
	COVID-19 hospitalization	-0.001	8.11E-01	218.674	2.22E-11
	SARS-CoV-2 infection	-0.002	6.96E-01	649.446	2.12E-81
Severe COVID-19	VTE	0.004	5.55E-01	57.052	8.02E-01
COVID-19 hospitalization		0.007	5.66E-01	190.929	4.71E-15
SARS-CoV-2 infection		0.022	2.78E-01	322.401	1.76E-41

Supplementary Table 10. Bi-directional Mendelian randomization analysis using GWAS summary statistics excluding data from UK Biobank for COVID-19. Estimates from inverse variance weighting were presented.

Direction	Number of SNPs	OR (95%CI)	P-value
VTE to severe COVID-19	99	1.08 (1.03-1.32)	7.84E-04
VTE to COVID-19 hospitalization	99	1.08 (1.04-1.12)	1.44E-05
VTE to SARS-CoV-2 infection	99	1.06 (1.03-1.09)	3.70E-05
Severe COVID-19 to VTE	88	0.97 (0.90-1.04)	3.87E-01
COVID-19 hospitalization to VTE	95	1.04 (0.92-1.16)	5.42E-01
SARS-CoV-2 infection to VTE	59	2.02 (1.23-3.30)	5.26E-03

Direction	No. of SNPs	OR(95%CI)	P-value
**weighted median**			
VTE to Severe Covid	98	1.10(1.03,1.17)	3.72e-03
VTE to Covid Hospitalization	99	1.07(1.02,1.11)	3.41e-03
VTE to SARS-Cov2 Infection	102	1.01(0.99,1.03)	4.60e-01
**MR-Egger**			
VTE to Severe Covid	98	1.14(1.02,1.27)	2.50e-02
VTE to Covid Hospitalization	99	1.11(1.02,1.21)	1.53e-02
VTE to SARS-Cov2 Infection	102	1.08(1.01,1.15)	2.83e-02
**MR-PRESSO**			
VTE to Severe Covid	98	1.12(1.07,1.17)	7.54e-06
VTE to Covid Hospitalization	99	1.08(1.05,1.12)	3.24e-06
VTE to SARS-Cov2 Infection	102	1.01(0.99,1.02)	4.25e-01
**MR-RAPS**			
VTE to Severe Covid	98	1.11(1.07,1.16)	7.93e-09
VTE to Covid Hospitalization	99	1.09(1.07,1.12)	8.53e-13
VTE to SARS-Cov2 Infection	102	1.02(1.01,1.04)	2.22e-05
**weighted mode**			
VTE to Severe Covid	98	0.97(0.82,1.15)	6.95e-01
VTE to Covid Hospitalization	99	1.03(0.96,1.10)	4.79e-01
VTE to SARS-Cov2 Infection	102	0.98(0.96,1.00)	8.88e-02

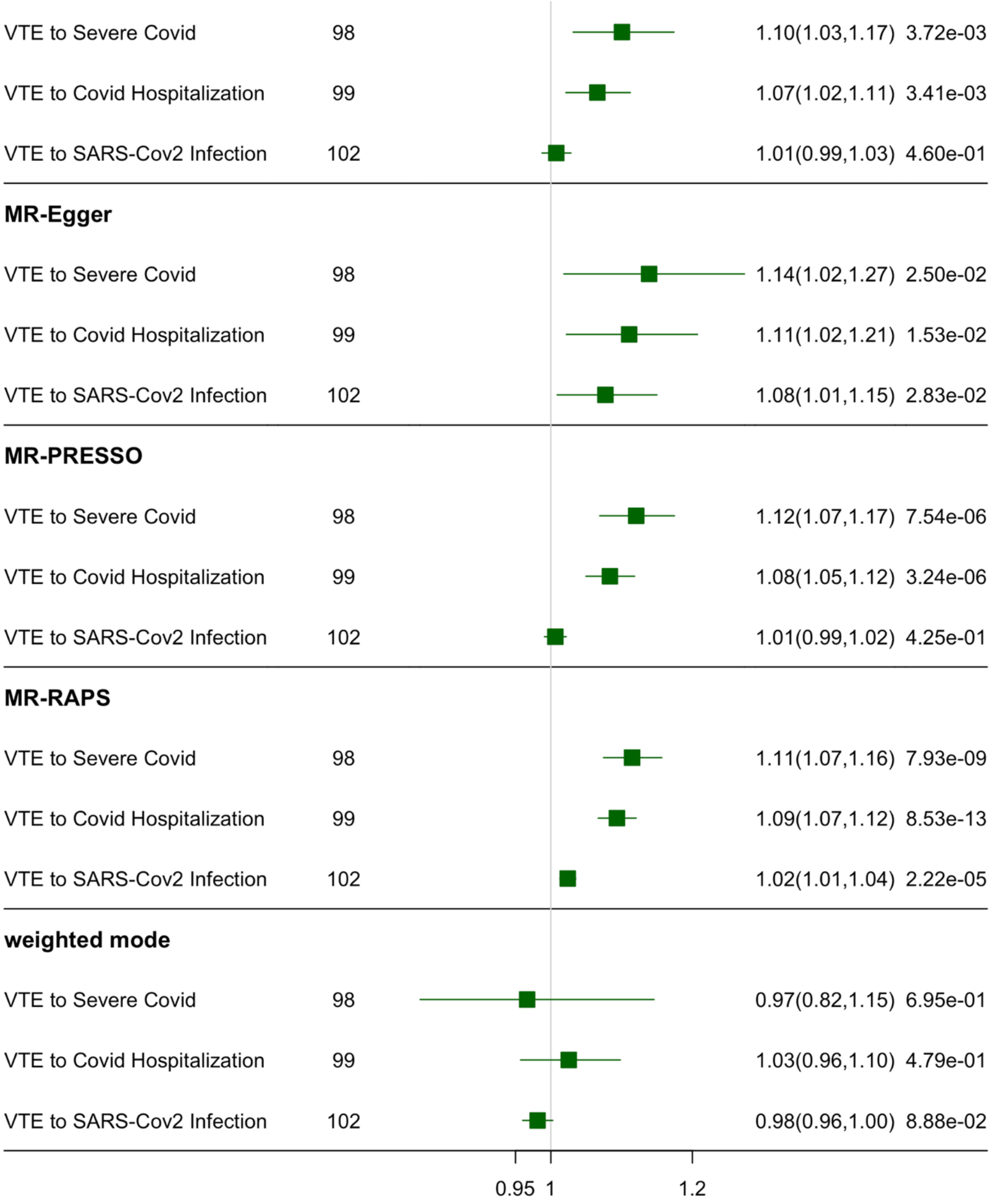

Supplementary Figure 1 MR analysis of genetically predicted VTE on the risk of COVID-19 related traits weighted median, MR-Egger, MR-PRESSO, MR-RAPS and weighted mode methods. The estimates are presented as odds ratios (OR) with 95% confidence intervals

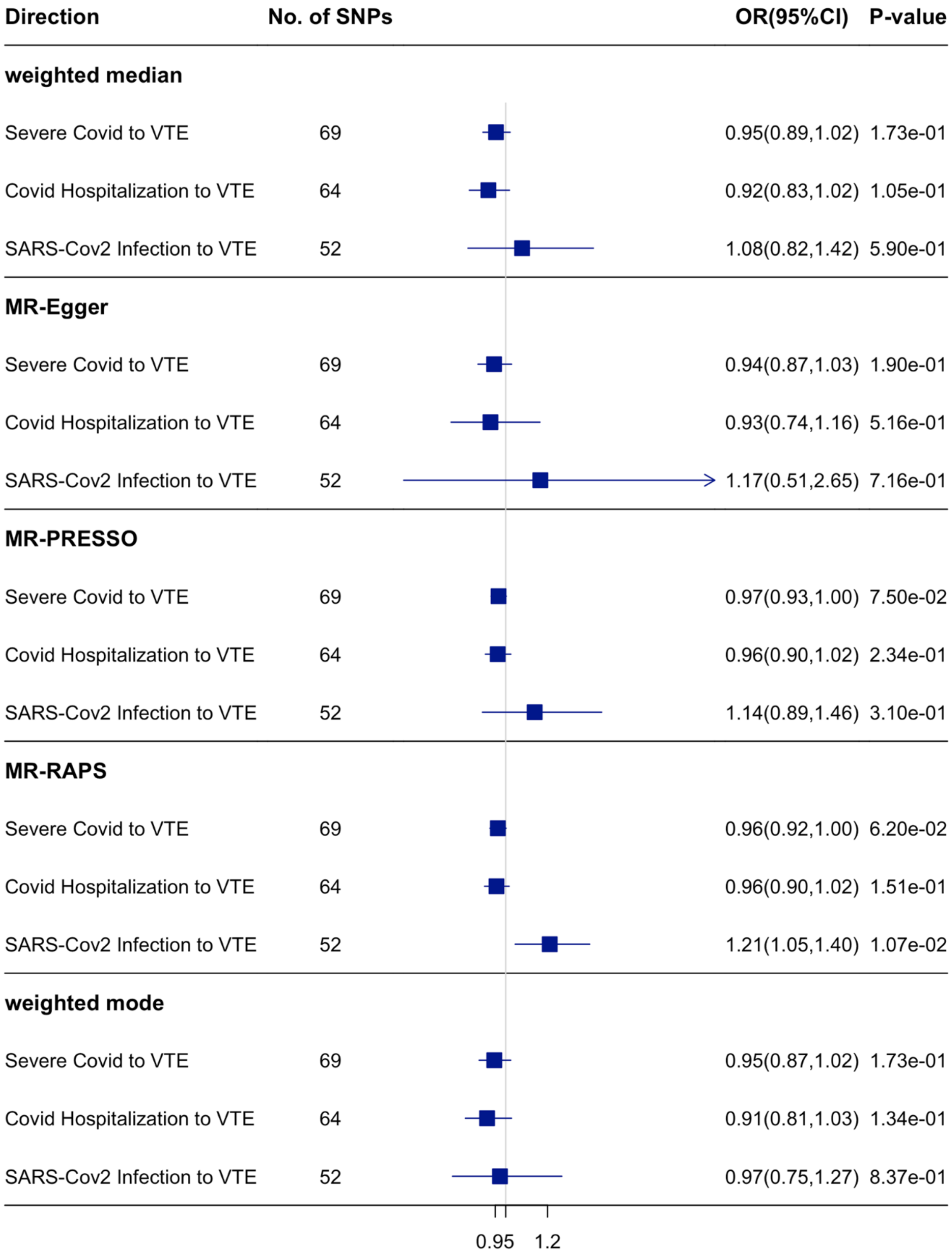

Supplementary Figure 2 MR analysis of genetically predicted COVID-19 related traits on the risk of VTE using weighted median, MR-Egger, MR-PRESSO, MR-RAPS and weighted mode methods. The estimates are presented as odds ratios (OR) with 95% confidence intervals (CI).

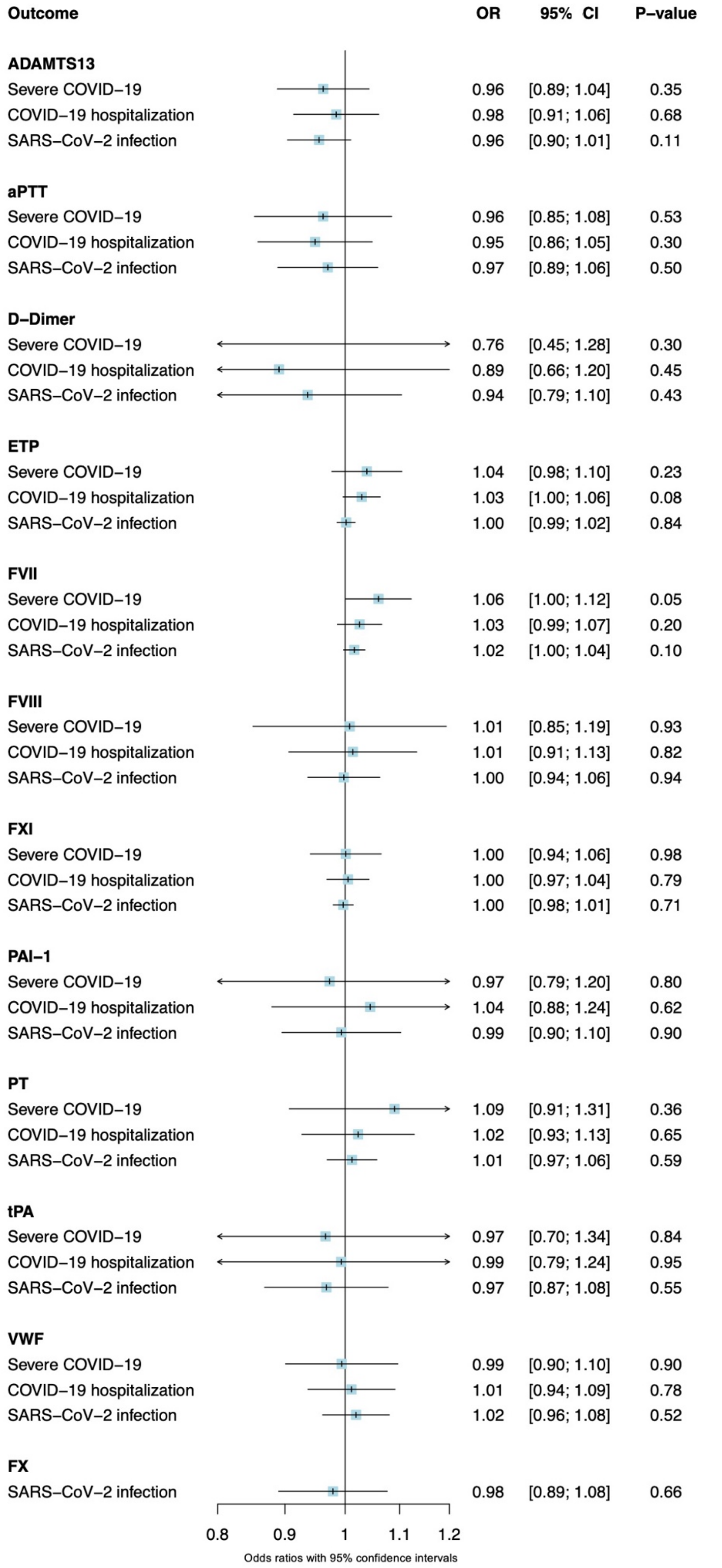

ADAMTS13, a disintegrin and metalloproteinase with a thrombospondin type 1 motif, member 13; aPTT, activated partial thromboplastin time; ETP, endogenous thrombin potential; FVII, Factor VII; FVIII, Factor VIII;FXI, Factor XI; PAI-1, plasminogen activator inhibitor-1; PT, prothrombin time; tPA, tissue plasminogen activator; VWF, von Willebrand factor; FX, Factor X.

Supplementary Figure 3 The Mendelian randomization associations of genetically predicted plasma levels of 12 coagulation factors with COVID-19 related traits. Estimates from inverse variance weighting and the corresponding 95% confidence interval (CI) were presented.
