# Supplementary material for: Genome-wide cross-trait analysis and Mendelian randomization reveal a shared genetic etiology and causality between COVID-19 and venous thromboembolism
Source: Commun Biol. 2023 Apr 21;6:441. doi: 10.1038/s42003-023-04805-2 (PMC10120502; doi:10.1038/s42003-023-04805-2)
Supplement: Supplementary file 3 — Description of Additional Supplementary Files [file 42003_2023_4805_MOESM3_ESM.pdf]

Description of Additional Supplementary Files

File name: Supplementary Data 1

Description: Genome-wide significant independent loci for COVID-19 related traits in GWAS of COVID-19 extracted based on COVID-19 Host Genetics Initiative. We used these loci to compare with our findings from MTAG, to check if we find novel loci.

File name: Supplementary Data 2

Description: Genome-wide significant independent loci for VTE

File name: Supplementary Data 3

Description: Genetic instruments for VTE used in the Mendelian randomization analysis

File name: Supplementary Data 4

Description: Genetic instruments for severe COVID-19 used in the Mendelian randomization analysis

File name: Supplementary Data 5

Description: Genetic instruments for COVID-19 hospitalization used in the Mendelian randomization analysis

File name: Supplementary Data 6

Description: Genetic instruments for SARS-CoV-2 infection used in the Mendelian randomization analysis

File name: Supplementary Data 7

Description: Source data for Figure 2

File name: Supplementary Data 8

Description: Source data for Figure 3(a)

File name: Supplementary Data 9

Description: Source data for Figure 3(b)

File name: Supplementary Data 10

Description: Source data for Figure 3(c)

File name: Supplementary Data 11

Description: Source data for Figure 4

File name: Supplementary Data 12

Description: Source data for Figure 5 and 6
