# Supplementary material for: Genome-wide cross-trait analysis and Mendelian randomization reveal a shared genetic etiology and causality between COVID-19 and venous thromboembolism
Source: Commun Biol. 2023 Apr 21;6:441. doi: 10.1038/s42003-023-04805-2 (PMC10120502; doi:10.1038/s42003-023-04805-2)
Supplement: Supplementary file 5 — Reporting Summary [file 42003_2023_4805_MOESM5_ESM.pdf]

nature portfolio

Corresponding author(s): Jie V Zhao, Zhonghua Liu

Last updated by author(s): Mar 25, 2023

Reporting Summary

Nature Portfolio wishes to improve the reproducibility of the work that we publish. This form provides structure for consistency and transparency in reporting. For further information on Nature Portfolio policies, see our Editorial Policies and the Editorial Policy Checklist.

Statistics

For all statistical analyses, confirm that the following items are present in the figure legend, table legend, main text, or Methods section.

n/a	Confirmed	
☐	☒	The exact sample size (*n*) for each experimental group/condition, given as a discrete number and unit of measurement
☐	☒	A statement on whether measurements were taken from distinct samples or whether the same sample was measured repeatedly
☐	☒	The statistical test(s) used AND whether they are one- or two-sided<br>*Only common tests should be described solely by name; describe more complex techniques in the Methods section.*
☐	☒	A description of all covariates tested
☐	☒	A description of any assumptions or corrections, such as tests of normality and adjustment for multiple comparisons
☐	☒	A full description of the statistical parameters including central tendency (e.g. means) or other basic estimates (e.g. regression coefficient) AND variation (e.g. standard deviation) or associated estimates of uncertainty (e.g. confidence intervals)
☐	☒	For null hypothesis testing, the test statistic (e.g. *F*, *t*, *r*) with confidence intervals, effect sizes, degrees of freedom and *P* value noted<br>*Give P values as exact values whenever suitable.*
☒	☐	For Bayesian analysis, information on the choice of priors and Markov chain Monte Carlo settings
☒	☐	For hierarchical and complex designs, identification of the appropriate level for tests and full reporting of outcomes
☒	☐	Estimates of effect sizes (e.g. Cohen's *d*, Pearson's *r*), indicating how they were calculated

Our web collection on statistics for biologists contains articles on many of the points above.

Software and code

Policy information about availability of computer code

Data collection	The data were downloaded from website (https://storage.googleapis.com/covid19-hg-public/freeze_7/results/20220403/pop_spec/sumstats/COVID19_HGI_A2_ALL_eur_leave23andme_20220403.tsv.gz; https://storage.googleapis.com/covid19-hg-public/freeze_7/results/20220403/pop_spec/sumstats/COVID19_HGI_B2_ALL_eur_leave23andme_20220403.tsv.gz; https://storage.googleapis.com/covid19-hg-public/freeze_7/results/20220403/pop_spec/sumstats/COVID19_HGI_C2_ALL_eur_leave23andme_20220403.tsv.gz; https://storage.googleapis.com/covid19-hg-public/freeze_7/results/20220403/leave_one_out/sumstats/COVID19_HGI_A2_ALL_leave_23andme_and_UKBB_20220403.tsv.gz; https://storage.googleapis.com/covid19-hg-public/freeze_7/results/20220403/leave_one_out/sumstats/COVID19_HGI_B2_ALL_leave_23andme_and_UKBB_20220403.tsv.gz; https://storage.googleapis.com/covid19-hg-public/freeze_7/results/20220403/leave_one_out/sumstats/COVID19_HGI_C2_ALL_leave_23andme_and_UKBB_20220403.tsv.gz; https://www.leelabsg.org/resources)
Data analysis	We used the following software and packages: R and R packages: R (version 4.0.5), TwoSampleMR (version 0.5.6), forestplot (version 2.0.1), mr.raps (version 0.2), dplyr (version 1.0.5), CMplot (version 3.6.2), corrplot (version 0.92), ggplot2 (version3.3.5), deTS (version 1.0), WebGestaltR (version 0.4.4), coloc (version 3.2-1), data.table (version 1.14.2), gprofiler2 (version 0.2.1)<br><br>Python and Python-based software: Python (version 2.7.5 & 3.6.3), LDSC (version 1.0.1), MTAG (version 1.0.8). The code were available upon reasonable request.

For manuscripts utilizing custom algorithms or software that are central to the research but not yet described in published literature, software must be made available to editors and reviewers. We strongly encourage code deposition in a community repository (e.g. GitHub). See the Nature Portfolio guidelines for submitting code & software for further information.

Data

Policy information about availability of data

All manuscripts must include a data availability statement. This statement should provide the following information, where applicable:

- Accession codes, unique identifiers, or web links for publicly available datasets
- A description of any restrictions on data availability
- For clinical datasets or third party data, please ensure that the statement adheres to our policy

Genetic associations with VTE were from the UK biobank GWAS results provided by Lee Lab (https://www.leelabsg.org/resources). Genetic associations with severe COVID-19, COVID-19 hospitalization and SARS-CoV-2 infection were obtained from COVID-19 host genetics consortium GWAS meta-analyses round 7, downloaded from https://storage.googleapis.com/covid19-hg-public/freeze_7/results/20220403/pop_spec/sumstats/COVID19_HGI_A2_ALL_eur_leave23andme_20220403.tsv.gz; https://storage.googleapis.com/covid19-hg-public/freeze_7/results/20220403/pop_spec/sumstats/COVID19_HGI_B2_ALL_eur_leave23andme_20220403.tsv.gz; https://storage.googleapis.com/covid19-hg-public/freeze_7/results/20220403/pop_spec/sumstats/COVID19_HGI_C2_ALL_eur_leave23andme_20220403.tsv.gz. In the MR sensitivity analysis, Genetic associations with severe COVID-19, COVID-19 hospitalization and SARS-CoV-2 infection excluding data from UK Biobank were obtained from https://storage.googleapis.com/covid19-hg-public/freeze_7/results/20220403/leave_one_out/sumstats/COVID19_HGI_A2_ALL_leave_23andme_and_UKBB_20220403.tsv.gz; https://storage.googleapis.com/covid19-hg-public/freeze_7/results/20220403/leave_one_out/sumstats/COVID19_HGI_B2_ALL_leave_23andme_and_UKBB_20220403.tsv.gz; https://storage.googleapis.com/covid19-hg-public/freeze_7/results/20220403/leave_one_out/sumstats/COVID19_HGI_C2_ALL_leave_23andme_and_UKBB_20220403.tsv.gz. Code book, and analytic code will be made available upon request pending application and approval by the corresponding author.

Human research participants

Policy information about studies involving human research participants and Sex and Gender in Research.

Reporting on sex and gender	We used summary statistics from GWAS of VTE and COVID-19. The GWAS of VTE was based on UK Biobank, a large cohort with 45.6% men. The summary statistics of COVID-19 were obtained from the COVID-19 host genetics initiative, which included several studies, the information on sex is currently not available.
Population characteristics	The summary statistics of VTE were based on UK Biobank, which recruited 502,713 people (aged 40-69 years, mean age 56.5 years, 45.6% men) from 2006 to 2010 in England, Scotland and Wales, 94% of self-reported European ancestry. The summary statistics of COVID-19 were obtained from the COVID-19 host genetics initiative, which included several studies. Here we only used people of European ancestry.
Recruitment	Not applicable for this study which was based on summary statistics of GWAS; no recruitment was conducted in this study.
Ethics oversight	The study is an analysis using publicly available summary data that does not require ethical approval.

Note that full information on the approval of the study protocol must also be provided in the manuscript.

Field-specific reporting

Please select the one below that is the best fit for your research. If you are not sure, read the appropriate sections before making your selection.

☒ Life sciences ☐ Behavioural & social sciences ☐ Ecological, evolutionary & environmental sciences

For a reference copy of the document with all sections, see nature.com/documents/nr-reporting-summary-flat.pdf

Life sciences study design

All studies must disclose on these points even when the disclosure is negative.

Sample size	The GWAS summary statistics for COVID-19 of European ancestry were provided by the COVID-19 host genetics initiative round 7 (https://www.covid19hg.org/results/, release date: April 08, 2022). We included three COVID-19 related traits: (1) Severe COVID-19, defined as COVID-19-confirmed individuals with very severe respiratory symptoms or those who died from the disease (up to 13,769 cases and 1,072,442 controls); (2) COVID-19 hospitalization defined as individuals who were hospitalized for related infection symptoms, with laboratory-confirmed SARS-CoV-2 infection (up to 32,519 cases and 2,062,805 controls); (3) SARS-CoV-2 infection defined as all individuals who reported positive (laboratory diagnosis, physician diagnosis or self-report) for SARS-CoV-2 infection (up to 122,616 cases and 2,475,240 controls). For VTE, we used summary statistics on the GWAS of VTE (3,900 cases and 369,592 controls of European ancestry) from the UK Biobank.
Data exclusions	No data were excluded.
Replication	We have conducted a replication MR analysis using GWAS summary statistics for COVID-19 excluding data from UK Biobank provided by the COVID-19 host genetics initiative round 7.

Randomization	Not relevant to this study because this is not a randomized controlled trial or experimental study.
Blinding	Not relevant to this study because this is not a randomized controlled trial or experimental study.

Reporting for specific materials, systems and methods

We require information from authors about some types of materials, experimental systems and methods used in many studies. Here, indicate whether each material, system or method listed is relevant to your study. If you are not sure if a list item applies to your research, read the appropriate section before selecting a response.

Materials & experimental systems

n/a	Involved in the study
☒	☐ Antibodies
☒	☐ Eukaryotic cell lines
☒	☐ Palaeontology and archaeology
☒	☐ Animals and other organisms
☒	☐ Clinical data
☒	☐ Dual use research of concern

Methods

n/a	Involved in the study
☒	☐ ChIP-seq
☒	☐ Flow cytometry
☒	☐ MRI-based neuroimaging
