# Supplementary material for: Genome-wide cross-trait analysis and Mendelian randomization reveal a shared genetic etiology and causality between COVID-19 and venous thromboembolism
Source: Commun Biol. 2023 Apr 21;6:441. doi: 10.1038/s42003-023-04805-2 (PMC10120502; doi:10.1038/s42003-023-04805-2)
Supplement: Supplementary file 1 — Peer Review File [file 42003_2023_4805_MOESM1_ESM.pdf]

nature portfolio

Peer Review File

Genome-wide cross-trait analysis and Mendelian randomization reveal a shared genetic etiology and causality between COVID-19 and venous thromboembolism

Open Access This file is licensed under a Creative Commons Attribution 4.0 International License, which permits use, sharing, adaptation, distribution and reproduction in any medium or format, as long as you give appropriate credit to the original author(s) and the source, provide a link to the Creative Commons license, and indicate if changes were made. In the cases where the authors are anonymous, such as is the case for the reports of anonymous peer reviewers, author attribution should be to 'Anonymous Referee' followed by a clear attribution to the source work. The images or other third party material in this file are included in the article's Creative Commons license, unless indicated otherwise in a credit line to the material. If material is not included in the article's Creative Commons license and your intended use is not permitted by statutory regulation or exceeds the permitted use, you will need to obtain permission directly from the copyright holder. To view a copy of this license, visit http://creativecommons.org/licenses/by/4.0/.

Reviewers' comments:

Reviewer #1 (Remarks to the Author):

Huang X et al have performed an interesting study exploring the genetics of VTE and COVID-19 related traits. Using known genome-wide signals at these traits they identified several shared risk loci and identified colocalized signals at ABO, ADAMTS13 and FUT2 genes, that may be involved in processes linked to coagulation and immune function. They further report on potential causal associations between VTE on COVID-19 related traits, while genetic instruments for SARS-CoV-2 infection was also associated with higher risk of VTE. The study is indeed timely and important, related to a significant outcome in COVID-19 patients. I have a few clarifications and suggestions that the authors could consider.

1) Besides evaluating the genetic correlations at only the known genome-wide hits for VTE and COVID related traits, it would be good to extend this in a genome-wide manner to fully understand overall genetic correlations and improve understandings of cross-trait genetic correlations. Similar evaluations have been recently performed, for example with psychiatric traits and education attainments (PMID: 31160569). Would the significance of coagulation and immune functions be diminished when evaluated in a genome-wide manner? Would there still be a significant enrichment in the lung if the analysis is performed genome-wide? A genome-wide evaluation of these cross-trait genetic correlations may serve to refine the biological pathways involved.

2) Figure 5 and 6 seem to seem to be the same figures.

3) The MR analysis requires additional details. Why include genetic variants that were not at genome-wide levels for VTE association (P < 1X10-5)? Would the associations still remain is only genome-wide signals were included? What was the P value cut-off in selecting signal genetic signals for COVID-19 traits?

Were any checks for pleiotropic SNPs performed during MR (MRegger slope evaluations, radial MR, etc)? Would similar association still remain if pleiotropic signals were excluded?

From supplementary fig2 only the MR-egger and MR-raps associations between SAR-CoV2 and VTE were significant. Would this be sufficient to draw conclusion on this possible causal effect?

Would there be any study related biases as effect estimates for VTE and COVID-19 traits have been drawn from prior studies that had utilized the same study dataset that has been used for the MR evaluations?

The authors could consider further evaluating the potentially causal signal between VTE and COVID-19 traits through a mediation framework.

Reviewer #2 (Remarks to the Author):

Huang et al use a variety of approaches to investigate the shared genetic architecture of VTE and COVID-19, including a Mendelian randomization analysis that provids evidence regarding the potential causal relationship between VTE and COVID-19. This is an interesting and important research question. There are some concerns outlined below that have implications on whether VTE is truly the causal agent, or whether other factors can instead explain the observed association (e.g. VWF).

Major comments

The authors identify several loci that they claim are associated with both COVID-19 and VTE, defined as having a multi-trait P-value < 5E-8 and single trait P-values < 0.05. This is an arbitrary defition, and some authors have used more stringent ones (e.g. PMC7860582 and others have used 0.005). Even more important than reconsidering what single-trait P-value to use is to consider colocolization as part of the defition. Loci that do not show co-locolization should not be considered pleiotropically associated with both.

The ABO and FUT2 loci are extremely pleiotropic, being associated with levels of countless proteins and with many quantitative and qualitative phenotypes. This is very well described in this preprint from UK Biobank: https://www.biorxiv.org/content/10.1101/2022.06.17.496443v1.full, but is also a well known phenomenon in the literature. Hence, ABO and FUT2 may not be valid genetic instruments, with ABO in particular having the ability to disrupt associations due to its strong association with VTE and many other phenotypes. Could the authors repeat the analysis after excluding ABO, and if necessary also FUT2?

Related to the comment above, ABO is strongly associated with von Willebrand Factor (VWF) and Factor VIII levels, which Mendelian randomization suggests are causal risk factors for VTE (PMC6438386). VWF has also been linked to COVID-19 severity. Could this be explored using a confounding or mediation framework?

Minor comments

Instead of using P(H4) for colocolization, the authors could consider using the conditional posterior probability that assumes association with both phenotypes: P(H4) / (P(H4) + P(H3)). This is also described in PMC7860582.

The ABO and ADAMTS13 locus are in the same genetic region. Were any steps besides distance-based pruning were taken to ensure that these two loci are statistically independent? Is there any evidence that the variant at the ADAMTS13 locus actually works through ADAMTS13?

Consider adding the weighted mode to the suite of MR sensitivity analyses.

We really appreciate the two reviewers for their valuable suggestions and comments. We have thoroughly considered all the comments and revised our manuscript, accordingly, especially redefined the shared loci and improved the Mendelian randomization analysis. Our responses to reviewers are given in a point-by-point manner below.

Reply to Reviewer #1:

Huang X et al have performed an interesting study exploring the genetics of VTE and COVID-19 related traits. Using known genome-wide signals at these traits they identified several shared risk loci and identified colocalized signals at ABO, ADAMTS13 and FUT2 genes, that may be involved in processes linked to coagulation and immune function. They further report on potential causal associations between VTE on COVID-19 related traits, while genetic instruments for SARS-CoV-2 infection was also associated with higher risk of VTE. The study is indeed timely and important, related to a significant outcome in COVID-19 patients. I have a few clarifications and suggestions that the authors could consider.

Response: Thank you very much for your positive remarks and constructive suggestions. We have carefully reviewed your suggestions and revised the manuscript accordingly. Please see the following for point-to-point responses.

1. Besides evaluating the genetic correlations at only the known genome-wide hits for VTE and COVID related traits, it would be good to extend this in a genome-wide manner to fully understand overall genetic correlations and improve understandings of cross-trait genetic correlations. Similar evaluations have been recently performed, for example with psychiatric traits and education attainments (PMID: 31160569). Would the significance of coagulation and immune functions be diminished when evaluated in a genome-wide manner? Would there still be a significant enrichment in the lung if the analysis is performed genome-wide? A genome-wide evaluation of these cross-trait

genetic correlations may serve to refine the biological pathways involved.

Response: We agree with the reviewer that estimating SNP-based genetic correlations should be in a genome-wide manner, and we actually performed the analysis in a genome-wide manner. We apologize that we may have not made it clear in the description of the methods in our initial submission. We performed genome-wide genetic correlation analysis, by calculating the linkage disequilibrium (LD) score across all SNPs. We have now highlighted this in the method section in the revised version to avoid any confusion.

We used Multi-Trait Analysis of GWAS (MTAG) to conduct cross-trait analysis to identify genome-wide significant associated loci between COVID-19 and VTE. MTAG is an approach for meta-analysis of summary statistics from genome-wide association studies (GWAS) of different traits.[1] The significance of the associated loci was evaluated in a genome-wide manner. We have also highlighted this in the method section. The results we found for enrichment in the lung were derived from tissue-specific expression analysis (TSEA) on the gene lists that were identified from cross-trait meta-analysis, which was based on a genome-wide analysis.

We revised the method as follows:

(Page 12, Method, Linkage disequilibrium score regression (LDSC) analysis, Line 273-275)

We added **"LDSC analysis was conducted to assess the heritability for a single trait and genome-wide genetic correlations between two traits, where genome-wide associations were used in the calculation."**

(Page 12-13, Method, Multi-Trait Analysis of GWAS (MTAG), Line 292-300)

We added **"We applied MTAG, an approach for meta-analysis of summary statistics from GWAS of different traits robust to sample overlap, to identify novel loci with strong signals for COVID-19, and to detect genome-wide significant loci**

between VTE and three COVID-19 traits.[1-3] MTAG can improve the effect estimates for each COVID-19 trait by incorporating the weighted sum of GWAS estimates for VTE. We also used MTAG to conduct genome-wide cross-trait meta-analysis, which utilizes sample size-weighted, fixed-effect model together with genetic covariance modeling from all sources to combine evidence of the genome-wide association between individual variants for VTE and COVID-19."

2. Figure 5 and 6 seem to seem to be the same figures.

Response: Thank you for pointing this out. We are very sorry that we placed the same two figures by mistake. We have now revised it. Please see Page 22, Figure 5 and 6.

3. The MR analysis requires additional details. Why include genetic variants that were not at genome-wide levels for VTE association (P < 1X10-5)? Would the associations still remain is only genome-wide signals were included? What was the P value cut-off in selecting signal genetic signals for COVID-19 traits?

Response: Thank you for your comments. In the previous analysis, we obtained genetic instruments for VTE from a published GWAS of VTE in Nature Genetics,[4] which selected genetic variants with $P < 1\times10^{-5}$ and with an R2 > 0.2 to increase the number of genetic instruments, and to improve genetic prediction. We used the same SNPs (297 SNPs) as the previous study and found that VTE was associated with higher risks of all COVID-19 related traits. These associations remain if we only used genome-wide significant ($P<5\times10^{-8}$) SNPs as genetic instruments for VTE.

We agree with the reviewer that using genome-wide significant ($P<5\times10^{-8}$) SNPs as genetic instruments would be better, which can minimize weak instrument bias.[5] We have now revised the criteria of identifying instruments in the bi-directional MR and updated the results.

We revised the method:

(Page 14, Method, Mendelian randomization (MR) analysis, Line 329-341)

"Genetic variants for VTE were obtained from a large GWAS of VTE restricted to Europeans (23,151 cases, 553,439 controls).[4] We extracted genome-wide significant ($P<5\times10^{-8}$) and uncorrelated ($r^2<0.2$) SNPs, and then excluded 6 SNPs in *ABO, FUT2, VWF* genes (Supplemental Table 13) as these gene regions have pleiotropic associations with multiple traits, including COVID-19. 107 SNPs has been selected as genetic instruments for VTE (Supplementary Table 13).[4] The genetic associations of these SNPs with COVID-19 related traits were obtained from COVID-19 HGI release 7 GWAS meta-analysis of European ancestry, as shown in study population. Using the same process of identifying instruments ($P<5\times10^{-8}$, $r^2<0.2$), and after excluding 2, 2, and 8 SNPs in *ABO, FUT2* or *VWF* genes, we obtained 87, 83 and 61 SNPs as instruments for severe COVID-19, COVID-19 hospitalization and SARS-CoV-2 infection respectively based on COVID-19 HGI release 7 GWAS meta-analysis (Supplementary Tables 14-16)."

We revised the results:

(Page 6, Results, Bi-directional MR analysis, Line 113-126)

"We found that genetically predicted VTE was positively associated with higher risk of all three COVID-19 related traits (OR= 1.11 for severe COVID-19, 95% CI: 1.06-1.17, P-value= 1.51×10^{-5}; OR=1.10 for COVID-19 hospitalization, 95% CI: 1.06-1.14, P-value =1.33×10^{-7}; OR=1.06 for SARS-CoV-2 infection, 95% CI: 1.04-1.09, P-value= 1.13×10^{-5}) (Figure 5)……. In the reverse direction, we found a positive association of genetically predicted SARS-CoV-2 infection with higher risk of VTE (OR=1.75, 95% CI: 1.18-2.58, P-value=4.92×10^{-3}), and null associations of genetically predicted severe COVID-19 or COVID-19 hospitalization with VTE (Figure 6)."

4. Were any checks for pleiotropic SNPs performed during MR (MRegger slope evaluations, radial MR, etc)? Would similar association still remain if pleiotropic

signals were excluded?

Response: Thank you for pointing this out. We have added MR-Egger intercept test in the analysis and showed its results in the results and supplementary table 11. MR-Egger intercept p values are all above 0.05, which did not indicate directional pleiotropy. We also used several other MR methods in the sensitivity analysis, including MR-PRESSO. MR-PRESSO can detect horizontal pleiotropic outliers, as well as correct for by removing these outliers.[6] The positive association of genetically predicted VTE with severe COVID-19 and COVID-19 hospitalization remained in the results of MR-PRESSO. We have highlighted this point in the result section.

We revised the results:

(Page 6, Results, Bi-directional MR analysis, Line 113-122)

We added **"We found that genetically predicted VTE was positively associated with higher risk of all three COVID-19 related traits (OR= 1.11 for severe COVID-19, 95% CI: 1.06-1.17, P-value= 1.51×10^{-5}; OR=1.10 for COVID-19 hospitalization, 95% CI: 1.06-1.14, P-value =1.33×10^{-7}; OR=1.06 for SARS-CoV-2 infection, 95% CI: 1.04-1.09, P-value= 1.13×10^{-5}) (Figure 5). MR-Egger intercept test did not indicate directional pleiotropy (Supplementary Table 11), which supported the validity of the findings. MR-PRESSO detected outliers in the associations of VTE with three COVID-19 traits (shown in Supplemental Table 13), and the positive associations of VTE with severe COVID-19 and COVID-19 hospitalization remained after removing these outliers (Supplementary Figure 1)."**

5. From supplementary fig2 only the MR-egger and MR-raps associations between SAR-CoV2 and VTE were significant. Would this be sufficient to draw conclusion on this possible causal effect?

Response: We found that the association of genetically predicted SARS-CoV-2 infection with higher risk of VTE was significant in the updated inverse variance weighted and

MR-RAPS results, but not significant in the weighted median, MR-Egger, MR-PRESSO and weighted mode methods, although they have consistent directions of associations. These lack of significance in some methods may be due to the wider confidence intervals. We agree, the significant associations may also occur by chance. Therefore, replication in a larger GWAS of SARS-CoV-2 infection is needed to draw conclusion on this causal association. We have now highlighted this point to the limitations of the study in the discussion section.

We revised the discussion:

(Page 10, Discussion, Line 222-237)

We added **"Fifth, genetic instruments for COVID-19 only capture a small proportion of variance. Although MR analysis provided unconfounded estimates, it is less precise than conventional observational studies. We observed a positive association of genetically predicted SARS-CoV-2 infection with VTE using inverse variance weighting and MR-RAPS, but the association included the null in weighted median, MR-Egger, MR-PRESSO and weighted mode, which may be due to the wider confidence intervals. Replication in a larger GWAS will be needed for validation."**

6. Would there be any study related biases as effect estimates for VTE and COVID-19 traits have been drawn from prior studies that had utilized the same study dataset that has been used for the MR evaluations?

Response: We obtained GWAS summary statistics for VTE from the UK Biobank and obtained GWAS summary statistics for COVID-19 related traits from multiple different cohorts including UK Biobank. Partial overlap of the sample may bias the estimates.[7] However, a simulation study has shown that bias from overlapping samples are of less concern in large biobank, such as UK Biobank.[8] In addition, we have conducted a replication analysis using GWAS summary statistics for COVID-19 excluding data from UK Biobank provided by the COVID-19 host genetics initiative round 7. We obtained

similar associations of VTE with three COVID-19 traits (shown in Table 1 as below). We have now added this analysis in the method section and showed the results in the result and discussion section.

We revised the method:

(Page 14, Method, Mendelian randomization (MR) analysis, Line 342-348)

*We added "***We used inverse variance weighted (IVW) analysis in the main analysis,[9] and used several other MR methods that are robust to pleiotropy, including the weighted median,[10] MR-Egger,[11] MR-PRESSO,[6] MR-RAPS,[12] and weighted mode[13] in sensitivity analysis. Considering sample overlap, we also repeated the bi-directional analysis in the sensitivity analysis using the GWAS summary statistics for COVID－19 excluding data from UK Biobank provided by COVID-19 HGI release 7 (https://www.covid19hg.org/results/).***"*

We revised the results:

(Page 6-7, Results, Bi-directional MR analysis, Line 125-128)

We added **"These results are robust to different MR methods (Supplementary Figure 2). And we obtained similar bi-directional associations between VTE and COVID-19 if we used COVID-19 HGI release 7 excluding data from UK Biobank (Supplementary Table 12)."**

We revised the discussion:

(Page 9, Discussion, Line 185-195)

We added **"Despite the statistical significance, our finding still needs to be interpreted with caution as we cannot exclude that this positive association may have occurred by chance. However, the positive association was also shown in a previous MR study using COVID-19 HGI release 5 data which showed that genetically predicted VTE was associated with higher risk of COVID-19 hospitalization and SARS-CoV-2 infection.[14] Using COVID-19 HGI release 7 with doubled sample size of release 5, we added by showing a positive association of**

genetically predicted VTE with higher risk of severe COVID-19. In addition, there are concerns that partial overlap of the samples may bias the estimates, however, we obtained consistent associations when we used COVID-19 HGI release 7 excluding data from UK Biobank.”

Supplementary Table 12. Bi-directional Mendelian randomization analysis using GWAS summary statistics excluding data from UK Biobank for COVID-19. Estimates from inverse variance weighting were presented.

Direction	Number of SNPs	OR (95%CI)	P-value
VTE to severe COVID-19	99	1.08 (1.03-1.32)	7.84E-4
VTE to COVID-19 hospitalization	99	1.08 (1.04-1.12)	1.44E-5
VTE to SARS-CoV-2 infection	99	1.06 (1.03-1.09)	3.70E-5
Severe COVID-19 to VTE	88	0.97 (0.90-1.04)	3.87E-1
COVID-19 hospitalization to VTE	95	1.04 (0.92-1.16)	5.42E-1
SARS-CoV-2 infection to VTE	59	2.02 (1.23-3.30)	5.26E-3

7. The authors could consider further evaluating the potentially causal signal between VTE and COVID-19 traits through a mediation framework.

Response: Thank you for your suggestion. It is well known that VTE is robustly associated with multiple coagulation factors,[15,16] and coagulation factors are involved in VTE. Coagulation factors are also risk factors for COVID-19, so these factors may be confounders or mediators between VTE and COVID-19 traits. However, the causal relationship of coagulation factors with COVID-19 is unclear. A previous Mendelian randomization study has explored the associations of 12 coagulation factors with COVID-19 severity based on the GWAS obtained from COVID-19 Host Genetics Initiative round 5,[17] and showed that only genetic predisposition to higher levels of VWF was associated with higher risk of COVID-19 severity. Given the previous study was based on a smaller GWAS of COVID-19, we performed an updated Mendelian randomization analysis using the most recent GWAS of COVID-19 from COVID-19

Host Genetics Initiative round 7, with much larger sample size than round 5. Results were shown in Figure 1 below. We found none of these genetically predicted coagulation factors were associated with the risk of COVID-19 related traits. In addition, we used MR analysis, which minimized confounding. And we have now excluded genetic instruments in ABO, FUT2 and VWF gene, which are involved in coagulation, and we obtained similar associations. So, these coagulation factors may not be potential confounders or mediators. Further studies are warranted to explore the existence of any other potential mediators. We have now illustrated this point in the discussion section.

We revised the discussion:

(Page 9, Discussion, Line 195-202)

We added **"Our study also provided timely evidence regarding the causal relationship between VTE and COVID-19 traits. …… We also considered that the observed associations may be mediated by coagulation factors, as VTE are associated with coagulation factors,[16] and coagulation is a risk factor of COVID-19.[18,19] Although a previous MR study on 12 coagulation factors and COVID-19 severity found that VWF was link to COVID-19 severity using COVID-19 HGI release 5,[20] we found null associations of these coagulation factors with three COVID-19 traits using the latest and much larger GWAS of COVID-19, COVID-19 HGI release 7 (Supplementary Figure 3). Further studies are needed to explore whether there are other potential mediators."**

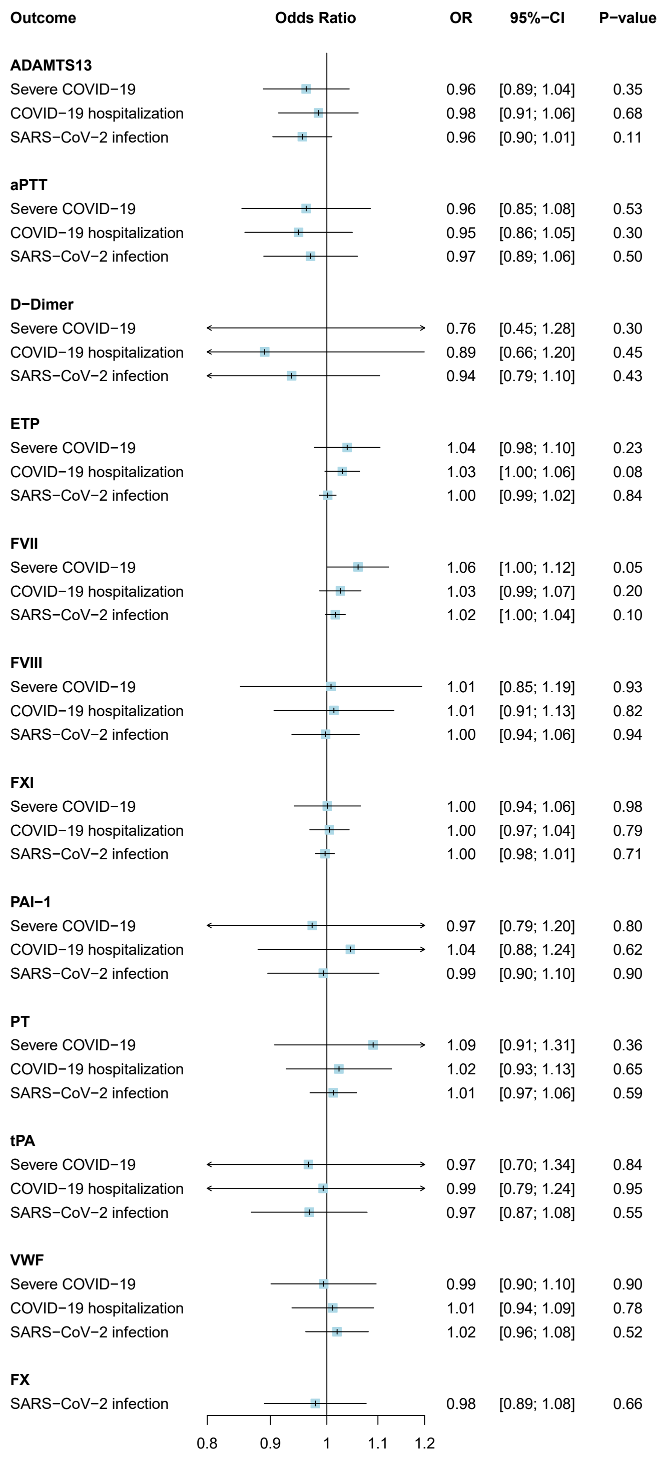

Supplementary Figure 3. The associations of genetically predicted plasma levels of 12 coagulation factors with COVID-19 related traits using Mendelian randomization. Estimates from inverse variance weighting were presented. ADAMTS13, a disintegrin and metalloproteinase with a thrombospondin type 1 motif, member 13; aPTT, activated partial

thromboplastin time; ETP, endogenous thrombin potential; FVII, Factor VII; FVIII, Factor VIII;FXI, Factor XI; PAI-1, plasminogen activator inhibitor-1; PT, prothrombin time; tPA, tissue plasminogen activator; VWF, von Willebrand factor; FX, Factor X.

Reply to Reviewer #2:

Huang et al use a variety of approaches to investigate the shared genetic architecture of VTE and COVID-19, including a Mendelian randomization analysis that provides evidence regarding the potential causal relationship between VTE and COVID-19. This is an interesting and important research question. There are some concerns outlined below that have implications on whether VTE is truly the causal agent, or whether other factors can instead explain the observed association (e.g. VWF).

Response: Thank you very much for your positive remarks. We have added and improved some analysis to address these concerns. Please see the following for point-to-point responses.

Major comments

1. The authors identify several loci that they claim are associated with both COVID-19 and VTE, defined as having a multi-trait P-value < 5E-8 and single trait P-values < 0.05. This is an arbitrary defition, and some authors have used more stringent ones (e.g. PMC7860582 and others have used 0.005). Even more important than reconsidering what single-trait P-value to use is to consider colocolization as part of the defition. Loci that do not show co-locolization should not be considered pleiotropically associated with both.

Response: Thank you for your constructive suggestions. We agree with the reviewer that loci showing co-localization gave more evidence that the two traits share a causal mechanism. We have now used a more stringent P-value ($P_{meta}<5\times10^{-8}$ in both traits and $P<5\times10^{-3}$ in single traits) and considers colocalization in the interpretation of the shared loci. More details about colocalization were added in the Supplementary Note.

We have now provided a more rigorous definition of shared loci in the method section.

The updated results showed that the cross-trait analysis identified 2 genetic loci associated with both VTE and severe COVID-19, 3 with both VTE and COVID-19 hospitalization and 4 with both VTE and SARS-CoV-2 infection, which were mapped to ABO, ADAMTS13 and FUT2 genes involved in coagulation function. Co-localization analysis showed that all these loci had the conditional posterior probability of colocalization above 0.8, indicating that loci identified in the meta-analysis are truly shared by COVID-19 and VTE.

We revised the method as follows:
(Page 13, Method, Multi-Trait Analysis of GWAS (MTAG), Line 296-301)
We added **"We also used MTAG to conduct genome-wide cross-trait meta-analysis, which utilizes sample size-weighted, fixed-effect model together with genetic covariance modeling from all sources to combine evidence of the genome-wide association between individual variants for VTE and COVID-19. Using cross-trait meta-analysis, we identified SNPs reaching genome-wide significance ($P_{meta}<5\times10^{-8}$) in both traits and suggestive significance (single trait $P<5\times10^{-3}$) in single traits.[21]"**

(Page 13, Method, Fine-mapping and co-localization analysis, Line 307-313)
We added **"For the loci associated with two traits, we used the Bayesian fine-mapping algorithm to identify a 99%-credible set of causal variants for each of the loci within 500kb of the index SNP.[22] Then, we conducted the co-localization analysis to check whether the loci identified in the cross-trait meta-analysis are causal variant shared between COVID-19 and VTE. In co-localization, we calculated the conditional posterior probability of colocalization ($P(H_4)/(P(H_3)+P(H_4))$).[23] If a SNP has the probability larger than 0.8, we labelled it as a co-localized genetic variant."**

We revised the result:

(Page 5, Results-Multi-Trait Analysis of GWAS (MTAG), Line 84-92)

This paragraph was revised to **"As shown in Table 1, we identified 2 genetic loci associated with both VTE and severe COVID-19, 3 with both VTE and COVID-19 hospitalization and 4 with both VTE and SARS-CoV-2 infection ($P_{meta} < 5\times10^{-8}$; single trait P $<5\times10^{-3}$). In line with previous studies,[24–29] we identified *ABO* gene and *FUT2* gene, which contributed to both VTE and COVID-19. The strongest association signals were localized on or near the *ABO* gene (index SNP: rs11244061 for severe COVID-19 and COVID-19 hospitalization; rs550057 for SARS-CoV-2 infection) at locus 9q34.2. We also identified *ADAMTS13*, which have not been reported yet. Figure 3 displays the Manhattan plots of these results."**

(Page 5, Results, Fine-mapping and colocalization analysis identify shared causal variants, Line 95-99)

This paragraph was revised to **"For each of loci associated with VTE and COVID-19, Supplementary Tables 5-7 listed all SNPs within 500 kb of these variants in the 99% credible sets. Co-localization analysis shows that all loci identified in cross-trait meta-analysis had the conditional posterior probability of colocalization >0.8, (Table 1), indicating that they are shared loci between the two traits."**

2. The ABO and FUT2 loci are extremely pleiotropic, being associated with levels of countless proteins and with many quantitative and qualitative phenotypes. This is very well described in this preprint from UK Biobank: https://www.biorxiv.org/content/10.1101/2022.06.17.496443v1.full, but is also a well known phenomenon in the literature. Hence, ABO and FUT2 may not be valid genetic instruments, with ABO in particular having the ability to disrupt associations due to its strong association with VTE and many other phenotypes. Could the authors repeat the analysis after excluding ABO, and if necessary also FUT2?

Response: Thank you for pointing this out. In the selection of genetic instruments for

VTE and COVID-19 in the MR analysis, considering the pleiotropy, we have now excluded SNPs in ABO and FUT2 genes, including rs78755596, rs7855466, rs11244051, rs9411377 in ABO associated with VTE; rs550057 in ABO and rs368565 in FUT2 associated with severe COVID-19; rs657152 in ABO and rs492602 in FUT2 associated with COVID-19 hospitalization; rs7469576, rs145526382, rs11244051, rs8176715, rs8176707, rs8176704, rs554833 in ABO and rs679574 in FUT2 associated with SARS-CoV-2 infection. Considering VWF is correlated with ABO blood types, we also excluded 2 SNPs in VWF associated with VTE (rs7135039 and rs183356). We obtained similar associations in the updated analysis.

We revised the method:

(Page 14, Method, Mendelian randomization (MR) analysis, Line 327-340)

We added **"We conducted a bidirectional MR analysis to assess the causal association between each COVID-19 related trait and VTE. Genetic variants for VTE were obtained from a large GWAS of VTE restricted to Europeans (23,151 cases, 553,439 controls).[4] We extracted genome-wide significant ($P<5\times10^{-8}$) and uncorrelated ($r^2<0.2$) SNPs, and then excluded 6 SNPs in *ABO, FUT2, VWF* genes (Supplemental Table 13) as these gene regions have pleiotropic associations with multiple traits, including COVID-19. 107 SNPs has been selected as genetic instruments for VTE (Supplementary Table 13).[4] The genetic associations of these SNPs with COVID-19 related traits were obtained from COVID-19 HGI release 7 GWAS meta-analysis, as shown in study population. Using the same process of identifying instruments ($P<5\times10^{-8}$, $r^2<0.2$), and after excluding 2, 2, and 8 SNPs in *ABO, FUT2* or *VWF* genes, we obtained 87, 83 and 61 SNPs as instruments for severe COVID-19, COVID-19 hospitalization and SARS-CoV-2 infection respectively based on COVID-19 HGI release 7 GWAS meta-analysis (Supplementary Tables 14-16)."**

We updated the results:

(Page 6, Results, Bi-directional MR analysis, Line 113-125)

This paragraph was revised to "**We found that genetically predicted VTE was positively associated with higher risk of all three COVID-19 related traits (OR= 1.11 for severe COVID-19, 95% CI: 1.06-1.17, P-value= 1.51×10^{-5}; OR=1.10 for COVID-19 hospitalization, 95% CI: 1.06-1.14, P-value =1.33×10^{-7}; OR=1.06 for SARS-CoV-2 infection, 95% CI: 1.04-1.09, P-value= 1.13×10^{-5}) (Figure 5). …… In the reverse direction, we found a positive association of genetically predicted SARS-CoV-2 infection with higher risk of VTE (OR=1.75, 95% CI: 1.18-2.58, P-value=4.92×10^{-3}), and null associations of genetically predicted severe COVID-19 or COVID-19 hospitalization with VTE (Figure 6).**"

3. Related to the comment above, ABO is strongly associated with von Willebrand Factor (VWF) and Factor VIII levels, which Mendelian randomization suggests are causal risk factors for VTE (PMC6438386). VWF has also been linked to COVID-19 severity. Could this be explored using a confounding or mediation framework?

Response: Thank you for your suggestion. It is well known that VTE is robustly associated with multiple coagulation factors,[15,16] and coagulation factors are involved in VTE. Coagulation factors are also risk factors for COVID-19, so these factors may be confounders or mediators between VTE and COVID-19 traits. However, the causal relationship of coagulation factors with COVID-19 is unclear. A previous Mendelian randomization study has explored the associations of 12 coagulation factors with COVID-19 severity based on the GWAS obtained from COVID-19 Host Genetics Initiative round 5,[17] and showed that only genetic predisposition to the levels of VWF was causally associated with the incidence of COVID-19 severity. Given the previous study was based on a smaller GWAS of COVID-19, we performed an updated Mendelian randomization analysis using the most recent GWAS of COVID-19 from COVID-19 Host Genetics Initiative round 7, with much larger sample size than round 5. Results were shown in Figure 1 above. We found none of these genetically predicted coagulation factors were associated with the risk of COVID-19 related traits. In addition, we used MR analysis, which minimized confounding. And we have now

excluded genetic instruments in ABO, FUT2, and VWF gene, which are involved in coagulation, and we obtained similar associations. So, these coagulation factors may not be potential confounders or mediators.

We also performed a Mendelian randomization analysis using genome-wide significant ($P<5\times10^{-8}$) and uncorrelated ($r^2<0.2$) SNPs (excluding SNPs on the ABO gene) obtained from the GWAS of PMC6438386 as genetic instruments for VWF and FVIII and found that genetically predicted plasma VWF and FVIII levels were not associated with the risk of all three COVID-19 traits. Results are shown in the following table.

The associations of genetically predicted plasma VWF and FVIII levels with COVID-19 related traits using Mendelian randomization. Estimates from inverse variance weighting were presented.

Exposure	***Outcome***	***OR (95%CI)***	***P-value***
VWF	*Severe COVID-19*	*0.65 (0.45-0.96)*	*3.04E-2*
	COVID-19 hospitalization	*0.86 (0.67-1.10)*	*2.21E-1*
	SARS-CoV-2 infection	*0.97 (0.88-1.12)*	*6.57E-1*
FVIII	*Severe COVID-19*	*0.70 (0.41-1.22)*	*2.10E-1*
	COVID-19 hospitalization	*0.98 (0.68-1.41)*	*9.06E-1*
	SARS-CoV-2 infection	*0.87 (0.72-1.06)*	*1.60E-1*

Although there was a marginal inverse association between VWF and severe COVID-19, it was not robust to other MR methods and did not remain after multiple testing correction. Therefore, there was little evidence that VWF and FVIII are confounders or mediators in the association of VTE with COVID-19. Further studies are needed to explore the existence of other potential mediators. We have now mentioned this point in the discussion section.

We revised the discussion:

(Page 9, Discussion, Line 195-202)

We added **"Our study also provided timely evidence regarding the causal**

relationship between VTE and COVID-19 traits. …… We also considered that the observed associations may be mediated by coagulation factors, as VTE are associated with coagulation factors,[16] and coagulation is a risk factor of COVID-19.[18,19] Although a previous MR study on 12 coagulation factors and COVID-19 severity found that VWF was link to COVID-19 severity using COVID-19 HGI release 5,[20] we found null associations of these coagulation factors with three COVID-19 traits using the latest and much larger GWAS of COVID-19, COVID-19 HGI release 7 (Supplementary Figure 3). Further studies are needed to explore whether there are other potential mediators."

Minor comments

6. Instead of using P(H4) for colocolization, the authors could consider using the conditional posterior probability that assumes association with both phenotypes: P(H4) / (P(H4) + P(H3)). This is also described in PMC7860582.

Response: Thank you for your suggestion. As recommended in PMC7860582, we have now redefined the variants as colocalized when the conditional posterior probability $P(H_4)/(P(H_3) + P(H_4) \geq 0.8$*. The results showed that all loci identified in the cross-trait meta-analysis met the colocalization criteria, which can be considered as shared loci between the two traits.*

We revised the method:

(Page 13, Method, Fine-mapping and co-localization analysis, Line 307-313)

We added **"For the loci associated with two traits, we used the Bayesian fine-mapping algorithm to identify a 99%-credible set of causal variants for each of the loci within 500kb of the index SNP.[22] Then, we conducted the co-localization analysis to check whether the loci identified in the cross-trait meta-analysis are causal variant shared between COVID-19 and VTE. In co-localization, we calculated the conditional posterior probability of colocalization ($P(H_4)/(P(H_3)+P(H_4))$).[23] If a SNP has the probability larger than 0.8, we labelled**

it as a co-localized genetic variant."

7. The ABO and ADAMTS13 locus are in the same genetic region. Were any steps besides distance-based pruning were taken to ensure that these two loci are statistically independent? Is there any evidence that the variant at the ADAMTS13 locus actually works through ADAMTS13?

Response: We used the PLINK clumping method to ensure SNPs associated with both traits found in the cross-trait meta-analysis are statistically independent, where SNPs that are in linkage disequilibrium with each other (r^2>0.01) within a distance of 500kb will be pruned. The SNPs with the lowest p-value are retained. We have now described this in the method section.

We found that rs149181677, the variant located on the ADAMTS13 gene, was shared between severe COVID-19, COVID-19 hospitalization and VTE. Although there is little evidence for the specific function of this variant, evidence from expression quantitative trait loci (eQTL) analysis showed that rs149181677 affects the expression of ADAMTS13 genes,[30] and gene expression is generally considered to affect protein levels, here ADAMTS13. Consistently, MR study supports genetically predicted ADAMTS13 is associated with COVID-19. We agree, currently we cannot fully confirm the link between this genetic variant with the protein ADAMTS13, as there is no study assessing the association between this genetic variant with ADAMTS13. Future studies are needed to test the link. We added this point in the limitation.

We revised the method:

(Page 13, Method, Multi-Trait Analysis of GWAS (MTAG), Line 295-304)

We added **"We also used MTAG to conduct <u>genome-wide</u> cross-trait meta-analysis, which utilizes sample size-weighted, fixed-effect model together with genetic covariance modeling from all sources to combine evidence of the <u>genome-wide association</u> between individual variants for VTE and COVID-19. <u>Using</u>**

cross-trait meta-analysis, we identified SNPs reaching genome-wide significance (P_{meta}<5×10^{-8}) in both traits and suggestive significance (single trait P<5×10^{-3}) in single traits.[21] We further applied the PLINK clumping function (r^2 threshold = 0.01, distance= 500kb) to ensure the independence of selected SNPs, i.e. SNPs in linkage disequilibrium that have a correlation >0.01 with the most significant SNP within a distance of 500 kb will be pruned.[31]"

We revised the discussion:

(Page 10-11, Discussion, Line 237-244)

We added "…… **Finally, although our study provides evidence of genetic correlation and genetic overlap between COVID-19 and VTE, the underlying biological mechanisms are still unclear. For example, we identified rs149181677, the variant located on the *ADAMTS13* gene, was shared between COVID-19 and VTE. Although this SNP was associated with the expression of *ADAMTS13* genes,**[30] **we cannot fully confirm the link between this genetic variant with the protein ADAMTS13, as there is no study assessing the association between this genetic variant with ADAMTS13. Future studies are warranted to test the link.**"

8. Consider adding the weighted mode to the suite of MR sensitivity analyses.

Response: Thank you for your suggestion. We have now added weighted mode in the MR sensitivity analysis. Results were shown in the Supplementary Figure 1 and 2.

1. Turley, P. *et al.* Multi-trait analysis of genome-wide association summary statistics using MTAG. *Nature genetics* 50, 229-237 (2018).
2. Chung, J. *et al.* Genome-wide association study of cerebral small vessel disease reveals established and novel loci. *Brain* 142, 3176-3189 (2019).
3. Guo, Y. *et al.* A genome-wide cross-phenotype meta-analysis of the association of blood pressure with migraine. *Nature Communications* 11, 3368 (2020).
4. Klarin, D. *et al.* Genome-wide association analysis of venous thromboembolism identifies new risk loci and genetic overlap with arterial vascular disease. *Nature genetics* 51, 1574-1579 (2019).
5. Davies, N.M., Holmes, M.V. & Davey Smith, G. Reading Mendelian randomisation

studies: a guide, glossary, and checklist for clinicians. *BMJ* 362, k601 (2018).
6. Verbanck, M., Chen, C.Y., Neale, B. & Do, R. Detection of widespread horizontal pleiotropy in causal relationships inferred from Mendelian randomization between complex traits and diseases. *Nat Genet* 50, 693-698 (2018).
7. Burgess, S., Davies, N.M. & Thompson, S.G. Bias due to participant overlap in two-sample Mendelian randomization. *Genet Epidemiol* 40, 597-608 (2016).
8. Minelli, C. *et al.* The use of two-sample methods for Mendelian randomization analyses on single large datasets. *International Journal of Epidemiology* 50, 1651-1659 (2021).
9. Burgess, S., Butterworth, A. & Thompson, S.G. Mendelian randomization analysis with multiple genetic variants using summarized data. *Genet Epidemiol* 37, 658-65 (2013).
10. Emdin, C.A., Khera, A.V. & Kathiresan, S. Mendelian Randomization. *Jama* 318, 1925-1926 (2017).
11. Bowden, J., Davey Smith, G. & Burgess, S. Mendelian randomization with invalid instruments: effect estimation and bias detection through Egger regression. *Int J Epidemiol* 44, 512-25 (2015).
12. Zhao, Q., Wang, J., Hemani, G., Bowden, J. & Small, D.S. Statistical inference in two-sample summary-data Mendelian randomization using robust adjusted profile score. *The Annals of Statistics* 48, 1742-1769, 28 (2020).
13. Hartwig, F.P., Davey Smith, G. & Bowden, J. Robust inference in summary data Mendelian randomization via the zero modal pleiotropy assumption. *Int J Epidemiol* 46, 1985-1998 (2017).
14. Xiang, Y., Chau, C.K.-L., Qiu, J., Rao, S. & So, H.-C. Exploring causal relationships between COVID-19 and cardiometabolic disorders: A bi-directional Mendelian randomization study. *medRxiv*, 2021.03.20.21254008 (2021).
15. Cushman, M., O'Meara, E.S., Folsom, A.R. & Heckbert, S.R. Coagulation factors IX through XIII and the risk of future venous thrombosis: the Longitudinal Investigation of Thromboembolism Etiology. *Blood* 114, 2878-83 (2009).
16. Sabater-Lleal, M. *et al.* Genome-Wide Association Transethnic Meta-Analyses Identifies Novel Associations Regulating Coagulation Factor VIII and von Willebrand Factor Plasma Levels. *Circulation* 139, 620-635 (2019).
17. Zhou, Y. *et al.* Coagulation factors and the incidence of COVID-19 severity: Mendelian randomization analyses and supporting evidence. *Signal transduction and targeted therapy* 6, 222-222 (2021).
18. Arachchillage, D.R.J. & Laffan, M. Abnormal coagulation parameters are associated with poor prognosis in patients with novel coronavirus pneumonia. *J Thromb Haemost* 18, 1233-1234 (2020).
19. Liao, D. *et al.* Haematological characteristics and risk factors in the classification and prognosis evaluation of COVID-19: a retrospective cohort study. *The Lancet Haematology* 7, e671-e678 (2020).
20. Zhou, Y. *et al.* Coagulation factors and the incidence of COVID-19 severity:

Mendelian randomization analyses and supporting evidence. *Signal Transduction and Targeted Therapy* **6**, 222 (2021).
21. Bone, W.P. *et al.* Multi-trait association studies discover pleiotropic loci between Alzheimer's disease and cardiometabolic traits. *Alzheimers Res Ther* **13**, 34 (2021).
22. Farh, K.K. *et al.* Genetic and epigenetic fine mapping of causal autoimmune disease variants. *Nature* **518**, 337-43 (2015).
23. Giambartolomei, C. *et al.* Bayesian test for colocalisation between pairs of genetic association studies using summary statistics. *PLoS Genet* **10**, e1004383 (2014).
24. Severe Covid, G.G. *et al.* Genomewide Association Study of Severe Covid-19 with Respiratory Failure. *The New England journal of medicine* **383**, 1522-1534 (2020).
25. Shelton, J.F. *et al.* Trans-ancestry analysis reveals genetic and nongenetic associations with COVID-19 susceptibility and severity. *Nat Genet* **53**, 801-808 (2021).
26. Roberts, G.H.L. *et al.* AncestryDNA COVID-19 Host Genetic Study Identifies Three Novel Loci. *medRxiv*, 2020.10.06.20205864 (2020).
27. Niemi, M.E.K. *et al.* Mapping the human genetic architecture of COVID-19. *Nature* **600**, 472-477 (2021).
28. Kousathanas, A. *et al.* Whole genome sequencing reveals host factors underlying critical Covid-19. *Nature* (2022).
29. Thibord, F. *et al.* Cross-Ancestry Investigation of Venous Thromboembolism Genomic Predictors. *medRxiv*, 2022.03.04.22271003 (2022).
30. Zhernakova, D.V. *et al.* Identification of context-dependent expression quantitative trait loci in whole blood. *Nature Genetics* **49**, 139-145 (2017).
31. Purcell, S. *et al.* PLINK: a tool set for whole-genome association and population-based linkage analyses. *Am J Hum Genet* **81**, 559-75 (2007).

REVIEWERS' COMMENTS:

Reviewer #1 (Remarks to the Author):

The authors have performed additional analyses to address the points that were raised and discussed additional and potential limitations of the study. I do not have other clarifications on the revised manuscript.

Reviewer #2 (Remarks to the Author):

The authors were responsive to both reviewers and addressed my concerns/comments.

REVIEWERS' COMMENTS:

Reviewer #1 (Remarks to the Author):

The authors have performed additional analyses to address the points that were raised and discussed additional and potential limitations of the study. I do not have other clarifications on the revised manuscript.

Thank you very much for your positive comment.

Reviewer #2 (Remarks to the Author):

The authors were responsive to both reviewers and addressed my concerns/comments.

Thank you very much for your positive comment.
